# Constructed Wetlands Suitability for Sugarcane Profitability, Freshwater Biodiversity and Ecosystem Services

**DOI:** 10.1007/s00267-022-01734-4

**Published:** 2022-10-21

**Authors:** Adam D. Canning, James C. R. Smart, Joshua Dyke, Graeme Curwen, Syezlin Hasan, Nathan J. Waltham

**Affiliations:** 1 Centre for Tropical Water and Aquatic Ecosystem Research (TropWATER), James Cook University, 1 James Cook Drive, Townsville, Queensland 4811 Australia; 2 School of Environment and Science, Griffith University, 170 Kessels Road, Nathan, Queensland 4111 Australia; 3 Australian Rivers Institute, Griffith University, 170 Kessels Road, Nathan, Queensland 4111 Australia

**Keywords:** Restoration, Flood reduction, Flow regulation, Fish habitat, Crop production

## Abstract

Freshwater ecosystems, such as wetlands, are among the most impacted by agricultural expansion and intensification through extensive drainage and pollution. There is a pressing need to identify ways of managing agricultural landscapes to ensure food and water security without jeopardising biodiversity and other environmental benefits. Here we examine the potential fish biodiversity and landholder financial benefits arising from the integration of constructed lagoons to improve drainage, flow regulation and habitat connectivity within a sugarcane dominated catchment in north Queensland, Australia. A hybrid approach was used, combining the findings of both fish ecological surveys and a financial cost-benefit analysis. We found that the constructed lagoons supported at least 36 native freshwater fishes (over half of all native freshwater fishes in the region), owing to their depth, vegetated margins, moderate water quality and high connectivity to the Tully River. In addition to biodiversity benefits, we estimated that surrounding sugarcane farms would have financially benefited from reduced flooding of cropland and the elevation of low-lying cropland with deposited spoil excavated from lagoon construction. Improved drainage and flow regulation allowed for improvement in sugarcane yield and elevated land increased gross margins from extending the length of the cane production cycle or enabling a switch from cattle grazing to cane production. Restoring or creating wetlands to reduce flooding in flood-prone catchments is a globally applicable model that could improve both agricultural productivity and aquatic biodiversity, while potentially increasing farm income by attracting payments for provision of ecosystem services.

## Introduction

With the global population expected to reach nine billion inhabitants by 2050, global food demand is expected to increase by 35–56% by 2050 from 2010 levels, consequently exacerbating tensions between land use for agriculture and habitat for biodiversity (van Dijk et al. 2021). Agricultural expansion and intensification can result in the loss of ecosystems and biodiversity, the eutrophication and sedimentation of aquatic ecosystems, increased greenhouse gas emissions, and altered hydrology (Tilman et al. 2001, 2002; Awuchi et al. 2020; Gaugler et al. 2020). Wetlands, including rivers and estuaries following RAMSAR convention (Ramsar Convention Secretariat 2016), are among the ecosystems most impacted by agricultural activities given their vulnerability to nutrient, sediment, and pesticide runoff (Buck et al. 2012; Ostrowski et al. 2021), water abstraction (Acreman et al. 2000), and to drainage and reclamation (Coleman et al. 2008). Global floodplain wetland loss is estimated at 95 km^2^/yr, though this loss is unlikely to slow or reduce under global human population predictions over the coming few decades (Coleman et al. 2008; Davidson 2014), with climate change set to exacerbate wetland loss and fragmentation across the globe (Segan et al. 2016). Despite this, approximately 10% of global animal biodiversity is found in freshwater ecosystems, which occupy <1% of Earth’s surface (Dudgeon 2019), with wetlands providing many ecosystem services (Barbier 2019; Davidson et al. 2019; Xu et al. 2020). There is, however, growing recognition of the need for the transformation of agricultural landscapes with both wetlands restoration and the application of agroecological principles, such as regenerative agricultural practices (LaCanne and Lundgren 2018; van Coppenolle and Temmerman 2019; Gliessman 2020). Large-scale wetlands construction and restoration are critical to advancing progress towards the UN Sustainable Development Goals (SDGs), including the universal provision of clean water access, the sustainable management of land and water ecosystems, and climate action (United Nations General Assembly 2015). Agroecology uses ecological principles to design and manage sustainable food systems, seeking to reconcile economic, environmental, and social dimensions (Gliessman 2020).

Like the rest of the world, Australia faces a legacy of degraded freshwater ecosystems, despite a small population and a relatively short 200 years of colonial urban, industrial, and agricultural development (Creighton et al. 2016). For example, between 2001 and 2017, the Great Barrier Reef (GBR) catchment experienced net loss of 740 ha (0.55% of 2001 extent) of coastal floodplain wetland. While contemporary loss has been relatively small, since European arrival, approximately 77,500 ha of palustrine wetland has been lost (~21.2% loss of pre-clear extent), much of which is from freshwater floodplain systems. Artificial/highly modified wetlands, however, have increased substantially between 2001 and 2017, increasing by 21,690 ha, much of which was created through the construction of tidal barrages (8299 ha) (Environmental Protection Agency 2005; Department of Environment and Science 2019). This loss of natural habitat, and the degradation of remaining habitat, is also reducing the GBR’s resilience to pressures from on-going pollutant runoff (Waterhouse et al. 2016; MacNeil et al. 2019; Adame et al. 2019a), and reducing habitat availability for species with freshwater life stages (Arthington et al. 2015; Adame et al. 2019a). Not only do coastal freshwater floodplain wetlands support diverse biological communities, but they also form part of important, broader, connected ecosystems, providing habitat for migratory species, flow regulation, and reduce sedimentation and nitrogen runoff (Bainbridge et al. 2009; Brodie and Waterhouse 2012; Waterhouse et al. 2016). While artificial/highly modified wetlands have been largely constructed for agricultural benefit, they still provide habitat for many wetland-dependent species and may be mitigating impacts of natural wetland loss (Canning and Waltham 2021).

Funding wetlands restoration and ensuring that it does not come at the expense of food production are two of the biggest barriers to large-scale wetland restoration (Waltham et al. 2020; Canning et al. 2021). While wetlands have been shown to improve food security directly through providing harvestable resources (e.g., fish, shellfish, and plants) (Cunningham 2015), food production can be improved indirectly through increased water security for irrigation and improved catchment drainage (Shennan and Bode 2002; Chen and Wong 2016). Leveraging on situations where wetland restorations also provide agricultural benefits (i.e., win-win scenarios) will be essential for realizing large-scale wetland restoration. In many situations, the financial benefits arising from wetland restoration need to outweigh the costs of restoring and maintaining the wetlands, and lost income. Where wetlands do not result in sufficient on-farm profit returns, financial incentive schemes, such as those paying for the provision of ecosystem services in the form or carbon sequestration, nutrient removal, or habitat provision, may help to ensure they are profitable at the farm scale (Banerjee et al. 2013; Sapkota and White 2020; Canning et al. 2021).

Instances where restored wetlands provide both agricultural and biodiversity benefits have primarily been due to the use of wetlands for growing rice, supporting livestock grazing, and improved water storage for irrigation (Verhoeven and Setter 2010; McIntyre et al. 2011; Peh et al. 2014). However, the benefits from using wetlands for drainage and flow regulation to improve both crop production and biodiversity simultaneously are rarely documented (Brander et al. 2013; Kadykalo and Findlay 2016).

On the floodplains of Australia’s wettest catchment (Tully-Murray), sugarcane farmers have grappled with the challenges of growing sugarcane on frequently flooded land, while supporting the health of the downstream Great Barrier Reef ecosystems. As part of Queensland’s $40 M (2001 AUD) Sugar Industry Infrastructure Program (hereafter ‘SIIP’), funded by the Queensland Government and the Australian Government, the Riversdale-Murray Valley Water Management Scheme (hereafter ‘the Riversdale-Murray Scheme’), completed in 2004, financially incentivised sugarcane farmers to construct wetlands across the Tully-Murray floodplain (Ernst and Young 2001). The Riversdale-Murray Scheme aimed to reduce cane inundation by flood waters and increase cane production across the Tully-Murray floodplain through the creation of arterial drainage and lagoon wetlands. Reduced inundation of mid-catchment cane land was achieved by directing water via preferential flow paths and draining it into ‘sump’ lagoons where the increased residence time then improved flow regulation and reduced flooding of downstream areas (Merrin; Karim et al. 2012, 2014). Lagoons were to have 100 m^2^ of surface area per hectare of their contributing catchment and a depth of up to 3 m to provide adequate detention time. Spoil excavated from a lagoon could be spread on adjacent low-lying areas to improve their productivity. Lagoons were also designed to be steep-sided to reduce weed growth, vegetated, and well-connected to the downstream river to provide on-farm fish habitat. As a result, a series of lagoons were created via the Riversdale-Murray Scheme for the specific purpose of providing both agricultural and biodiversity benefits (Merrin).

With over 15 years of wetland maturation and stakeholder hindsight, the Riversdale-Murray Scheme provides an opportunity to evaluate the impacts of catchment-integrated wetlands, designed primarily for improved drainage and flow regulation, on sugarcane profitability, fish biodiversity, and an array of ecosystem services more broadly. In this study, we aimed to:

1. Quantify the fish biodiversity, and associated water quality, provided by scheme-funded wetlands;
2. Estimate the return on investment and the benefit to cost ratio from the perspective of a representative landholder investing in wetland construction via the Riversdale-Murray Scheme; and
3. Identify the range of final ecosystem services potentially provided by the Scheme-funded wetlands.

## Methods

Our study involved a mixed-method approach, whereby ecological and economic analyses were carried out concurrently. The ecological assessments were primarily informed by field surveys and laboratory analysis; while the economic analyses were primarily informed by financial records, landholder surveys, and climatic and satellite remote sensing data.

### Study Area

**Fig. 1:**
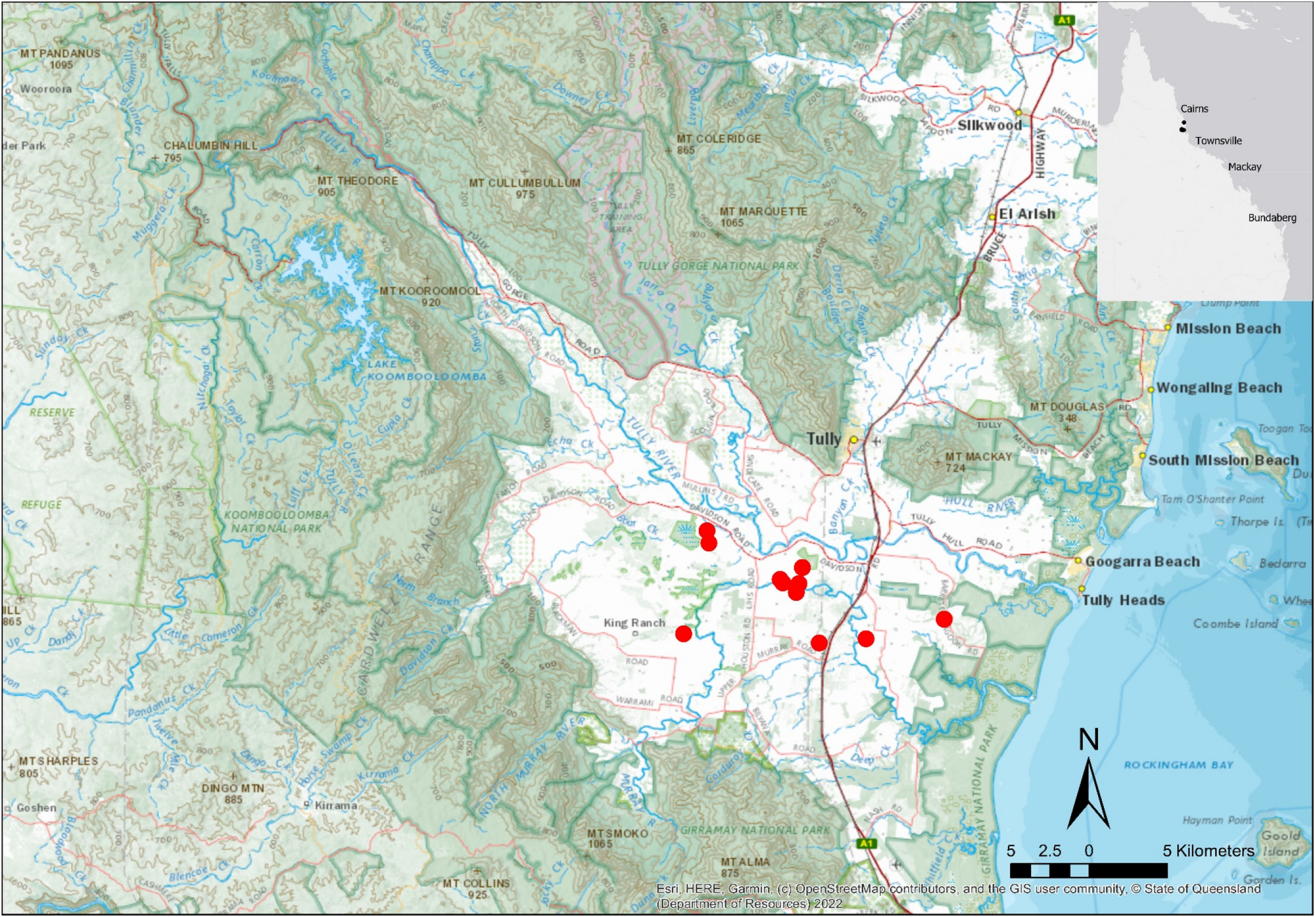
Locations of created wetlands sampled within the Tully-Murray catchment, Queensland. Topographic base map sourced from State of Queensland (Department of Resources) (2021)

The Riversdale-Murray scheme occurred across the Tully-Murray floodplain (Queensland), approximately 140 km south of Cairns and 200 km north of Townsville. Tully is often the wettest region of Australia, with an annual average rainfall of greater than 4000 mm (Fig. 1). The Tully and Murray rivers begin with headwaters in mountainous tropical rainforest protected as several National Parks, approximately 50 km (Euclidian distance) from the coastline. The lowland reaches drain flat agricultural land that primarily supports sugarcane farming. Across the Tully River—Murray River Floodplains there is approximately 43.3 km^2^ of DIWA nationally important wetlands, largely comprised of palustrine and riverine systems, covering 30.9 km^2^ and 9.7 km^2^, respectively (Environmental Protection Agency 2005; Department of Environment and Science 2019).

### Fish Biodiversity and Water Quality

Fish assemblages were surveyed at twelve lagoons in October 2019 and repeated at nine lagoons in June 2020 (Fig. 1), to assess community composition and structure, from which species richness was estimated. The GPS locations and landholder details are withheld to respect privacy, though dimensions summarized in Table 1. Fish assemblages were surveyed using a Smith-Root 2.5 GPP generator boat-mounted electrofishing unit. Surveys involved a single pass navigation of the entire edge habitat (where most fish reside) and zig-zagging through the middle until no new species were observed after 30 min of sampling. Pusey et al. (1998) demonstrated, in the nearby Johnstone and Mary Rivers, that most species can be detected within a single pass. The electrofishing unit supplied a pulsed direct current at 30–60 Hz with a duty cycle between 10 and 25%, settings were adjusted depending on a site’s electrical conductivity, depth and species composition. After assessing electrical conductivity, the output was gradually increased until fish were mildly stunned without incurring physical harm. Fish surveys were completed in accordance with the *Queensland Animal Care and Protection Act 2001*, and JCU animal ethics permit number A2178. All fish were measured (standard length in mm) and identified according to Allen et al. (2002). Sampling was non-destructive with all fish returned to the water, apart from non-native species which were retained and euthanised in accordance with Queensland’s *Biosecurity Act 2014*.

**Table 1:** Descriptive statistics of scheme-funded lagoons as catalogued in Riversdale-Murray Valley Water Board archival data, along with details for a mock-landholder considered representative of the participants involved used for analysis purposes

Data	Mean	Median	Max	Min	Representative
Surface area (ha)	0.41	0.25	2.55	0.02	0.3
Maximum depth (m)	3.24	4	5	0.7	3.5
Volume (m^3^)	8225	4072	25,000	500	10,500
Total Construction cost $ (2003 AUD)^a^	13,019	8000	30,000	1000	^b^20,053
Construction cost to farmer $ (2003 AUD)^a^	4340	2667	10,000	333	^c^6684
Area of soil spreading (ha)	1.45	1.11	4.09	0.38	

^a^2003 AUD$ can be converted to 2019 AUD$ by multiplying by 1.49. The conversion factor is the ratio of the index numbers for Dec 2003 to Dec 2019 reported in the Quarterly Consumer Price Index for Brisbane, Queensland (Australian Bureau of Statistics 2020)

^b^Total cost of construction obtained via fitted regression Eq. (1) in the main text

^c^Farmer contribution is 33% of total cost. The remainder is covered by the scheme subsidy

At each site, grab samples were collected to assess for an array of nutrients, sediment, and toxicants from three random locations (Tables S1 & S2). At the same time, a calibrated multi-parameter sensor (Quanta, Hydrolab Corporation) was used to profile (at 0.25 m intervals, down to 3.50 m where possible) the pH, electrical conductivity (EC), temperature and dissolved oxygen (Table S3). Collection was made with the vessel facing into the current, at 15–30 cm subsurface, with sterile containers and gloves. Care was taken to ensure that the bottom sediment was not disturbed and that surface films were not collected, in accordance with the Queensland sampling manual (Department of Environment and Science 2018). While animal movement may affect bottom water quality surveys, having three random replicate samples enables some encapsulation of variability. Water samples for filterable nutrients were syringe-filtered on site with an unused disposable plastic 60 mL syringe, 0.45μm Sartorius minisart filters, and were kept on ice until late freezing within 8 h, and eventually processed at a NATA accredited laboratory (American Public Health Association (APHA) et al. 2005; DERM 2009).

A calibrated multi-probe data logger (Hydrolab HL7, OTT HydroMet Ltd) was deployed in the near-surface water layer (0.2 m below the surface) at wetland sites to measure diel periodicity (cycling) of these physico-chemical parameters (water temperature, dissolved oxygen, conductivity, pH) at 20 min intervals (Table S4). All loggers remained at a site overnight to measure diel patterns.

Nutrient, sediment, and toxicant samples were compared against the relevant water quality objectives prescribed by the *Environmental Protection (Water and Wetland Biodiversity) Policy 2019: Tully River, Murray River and Hinchinbrook Island Basins Environmental Values and Water Quality Objectives*, or the Australian and New Zealand Environment and Conservation Council (2000) trigger values, where available, for parameters not covered by the policy. While dissolved oxygen concentrations were assessed against the north Australian default guideline values by Butler and Burrows (2007).

### Landholder Financial Assessment

Here we evaluate the return on investment that a mock-landholder, considered representative of the participants, obtained from the costs incurred in constructing a representative scheme-subsidised lagoon on a medium-sized cane farm. Using a mock-landholder allows for a generally applicable financial assessment while avoiding the need to disclose real landholder financial information, following Human Ethics approval requirements. Prior to the scheme, frequently flooded land only supported light cattle grazing or sugarcane production with a maximum of two ratoon crops. Return on the private investment in lagoon construction is calculated from the estimated financial benefits of improved drainage across the farm as a whole, and the increased gross margins under two scenarios arising from elevating adjacent land with dredge spoil: (1) conversion of cattle grazing to cane growing, or (2) lengthening of the existing cane production cycle from two to four ratoon crops after the initial plant cane crop.

The cost incurred by the landholder comprises 33% of the construction cost of their on-farm drainage lagoon (the remaining 67% of construction costs being covered by the scheme subsidy), plus any on-going maintenance costs. Landholder’s costs were incurred as a combination of in-kind and cash costs. Data on construction and maintenance costs were sourced from archived Riversdale-Murray Valley Water Board lagoon and drainage designs, farm compliance plans, and from semi-structured stakeholder interviews (*N* = 5, Note S1). Benefits, in terms of gross margin, were estimated using the online Farm Economic Analysis Tool (FEAT) developed by the Queensland Department of Agriculture and Fisheries (State of Queensland Department of Agriculture and Fisheries 2020).

#### Construction and Maintenance Costs

Construction costs for individual cost elements in farm-scale drainage designs were extracted (in 2003 AUD $) from archived Riversdale-Murray Valley Water Board farm compliance plans for 44 drainage features (drainage lagoons and excavated silt traps) on 16 properties. Data extracted was used to estimate a function for the total cost (Scheme subsidy plus farmer contribution, with the farmer contribution reported in 2003 AUD $ as a combination of in-kind and cash) of constructing farm-scale drainage lagoons using the linear regression model in below equation:

1 $$Cost_i = \alpha + \beta _1Vol\_Excavated_i + \beta _2Vol\_Excavated\_Sq_i + \beta _3SurfaceArea_i + \varepsilon _i$$

where the subscript *i* is an index for lagoon; *α* is a constant term; *β*_1_, *β*_2_ and *β*_3_ are coefficients to be estimated for the cost drivers: volume excavated (m^3^) as linear and squared variables, (*Vol*_*Excavated* and *Vol*_*Excavated*_*Sq*) and lagoon surface area in (m^2^) (*SurfaceArea*), respectively. *ε*_1_ is the error term which is assumed to be normally distributed. Equation (1) was then used to estimate the cost of constructing a representative lagoon with a size of 0.3 ha, (Table 1), escalating the estimated cost to 2019 AUD $ using the Quarterly Consumer Price Index for Brisbane, Queensland (Australian Bureau of Statistics 2020).

Maintenance costs were estimated from the semi-structured farmer interviews (*N* = 5), in which farmers were asked to quantify the operations undertaken and the annual costs incurred in maintaining their scheme-funded wetland (Note S1). Maintenance costs for the representative wetland were based on these responses, again escalated to 2019 AUD ($) using the Quarterly Consumer Price Index for Brisbane, Queensland.

#### Drainage

The extent and frequency of cane inundated by water was derived using Digital Earth Australia’s ‘Water observations from Space’ dataset from 1986 to 2019 (Geoscience Australia; Mueller et al. 2016). This dataset reports the number of times over a specified period that 25 m × 25 m pixels have been (i) observed and (ii) deemed to have been covered by water when viewed with multi-band imaging from Landsat satellites every 16 days. Cane land extent (13,606 ha) was derived from the Queensland Land Use Mapping Program (QLUMP) dataset across the Riversdale-Murray Valley Drainage Scheme area (Queensland Government 2015). This dataset was split into pre- (1986–2004) and post-drainage scheme (2005–2019) segments to estimate the proportion of cane land in the scheme area for which inundation time reduced after implementation of the Riversdale-Murray Scheme. For each segment, the frequency of pixel observation, and the frequency of those observations in which pixels were ‘wet’ were calculated. The frequency of both observations was then normalised per year to adjust for the different durations of the pre- and post-drainage datasets. The percentage of wet observations per pixel, pre- and post-scheme establishment, was then used to estimate the area of cane land across which inundation duration reduced or increased. As potential changes in rainfall may have influenced inundation patterns, we also used a Welch two-sample t-test to compare mean annual rainfall between the two periods. Rainfall data (1986–2019) was sourced from the Bureau of Meteorology (BOM) rainfall gauge at Tully Sugar Mill (monitoring station number 32042), approximately 10 km north-east of the Scheme area (Figure S1). Previous hydrological modelling has also demonstrated the efficacy of the lagoons and drainage network in altering flood regimes (Karim et al. 2012, 2014).

#### Economic Benefit from Reduced Duration of Inundation

The original business case for the Riversdale-Murray Scheme estimated the planned drainage improvements would reduce average cane losses from inundation of cane land by between 1.7 and 3.4 tonnes per hectare (Merrin). The increase in gross margin per hectare arising from a given increase in cane yield can be estimated using below equation (Canegrowers 2020a):

2 $$GM_{inc} = \left( {\left( {P_s \times 0.009 \times \left( {CCS - 4} \right)} \right) + C_c - C_h} \right) \times Y_{inc}$$

where *GM*_*inc*_ is the increment to gross margin ($/ha), *P*_*s*_ is the market price of sugar ($/tonne of sugar), *CCS* is the percentage sugar content per tonne of cane harvested (expressed as a %, i.e., ‘13’ denotes 13%), *C*_*c*_ is the constant term in the cane price formula ($/tonne of cane), *C*_*h*_ is the harvesting cost ($/tonne of cane), and *Y*_*inc*_ is the increment to yield (tonnes/ha) (Canegrowers 2020b). Equation (2) was applied to calculate the increment to gross margin for a medium-sized cane farm (150 ha) in the Murray catchment for cane yield increments of 1.7 and 3.4 tonnes per hectare, using the default FEAT market price of sugar, constant term, and cane harvesting cost for 2015 (State of Queensland 2016), escalated to 2019 AUD ($) using the Quarterly Consumer Price Index for Brisbane.

#### Farm Economic Analysis Tool (FEAT)

The economic benefit from the two productivity improvement scenarios is calculated from the increased gross margin that follows from either: (i) extension of the cane rotation from plant cane plus two ratoons to plant cane plus four ratoons, or (ii) from the switch from cattle fattening to cane production on a plant cane plus four ratoon production cycle. Gross margins from cane are estimated using FEAT (State of Queensland Department of Agriculture and Fisheries 2020), starting from the default 2015 activity and cost profile for cane production in the Tully catchment (the Riversdale-Murray Valley scheme was directly adjacent to this catchment) (State of Queensland 2016), but using cane yields predicted by the APSIM (Agricultural Production Systems Simulator) cane production simulation software for the climate zone, soil type and soil permeability surrounding a representative Riversdale-Murray Valley lagoon (Keating et al. 2003). In estimating the gross margins from cane production, the FEAT default region-specific assumptions and costs (in 2015 AUD) incurred in implementing the full suite of cane production activities were used initially (Table S5), before escalating the resulting gross margin to 2019 AUD ($) using the Quarterly Consumer Price Index for Brisbane. Gross margins from cattle fattening on poorly draining alluvial loam soil in the Tully-Murray catchment were drawn from Roebeling et al. (2007), escalated to 2019 AUD ($) using the Quarterly Consumer Price Index for Brisbane.

#### Return on Investment

The return on investment a representative landholder would be expected to obtain from the costs they incurred in constructing and maintaining a representative scheme-subsidised lagoon on their cane farm was calculated using discounted cash flow analysis over the 15-year period 2019 to 2034 inclusive. An annual real discount rate of 5% per annum is assumed, in common with much of the literature e.g., Alluvium (2019). For this analysis, all costs and benefits were expressed in 2019 AUD ($). The net present value achieved over the analysis period is given by below equation:

3 $$NPV = \mathop {\sum}\limits_{i = 0}^{15} {\frac{{B_t}}{{\left( {1 + r} \right)^t}}} - \mathop {\sum}\limits_{t = 0}^{15} {\frac{{C_t}}{{\left( {1 + r} \right)^t}}}$$

where *NPV* denotes net present value, *t* denotes the year within the 15-year analysis time span (commencing in 2019), *B*_*t*_ denotes the monetary benefit arising in year *t*, *C*_*t*_ denotes the monetary cost incurred in year *t*, and *r* denotes the real discount rate (5% per annum). The benefit to cost ratio (BCR) and internal rate of return (IRR) are calculated. The IRR delivered by the representative farmer’s investment in lagoon construction and maintenance was calculated as that discount rate which when inserted in Eq. (3) reduces *NPV* to zero. IRR reports the return on investment delivered by lagoon construction.

### Identification of Final Ecosystem Services

Final ecosystem services are the benefits arising from an ecosystem that flow directly to and are directly used by humans (Boyd and Banzhaf 2007; Johnston and Russell 2011; Mace et al. 2012). Final ecosystem services, and their classification systems, are used to allow more accurate and consistent definitions of ecosystem services, improve communication, and allow more seamless integration with national accounting (Johnston and Russell 2011; Wong et al. 2015; Finisdore et al. 2020). Using the Common International Classification of Ecosystem Services framework (Haines-Young and Potschin 2012), we sought to identify other ecosystem services that are potentially supported by the scheme-funded lagoons that remain as hypotheses with various levels of examination. This exercise only aimed to hypothesize potential services and did not attempt to quantify the benefit of each service. We also assigned pedigree scores for each hypothesized service to indicate our confidence in a service being provided, ranging from 1 (low confidence) to 4 (total confidence), in line with those proposed by Costanza et al (Costanza et al. 1992).

## Results

### Fish Biodiversity and Water Quality

**Fig. 2:**
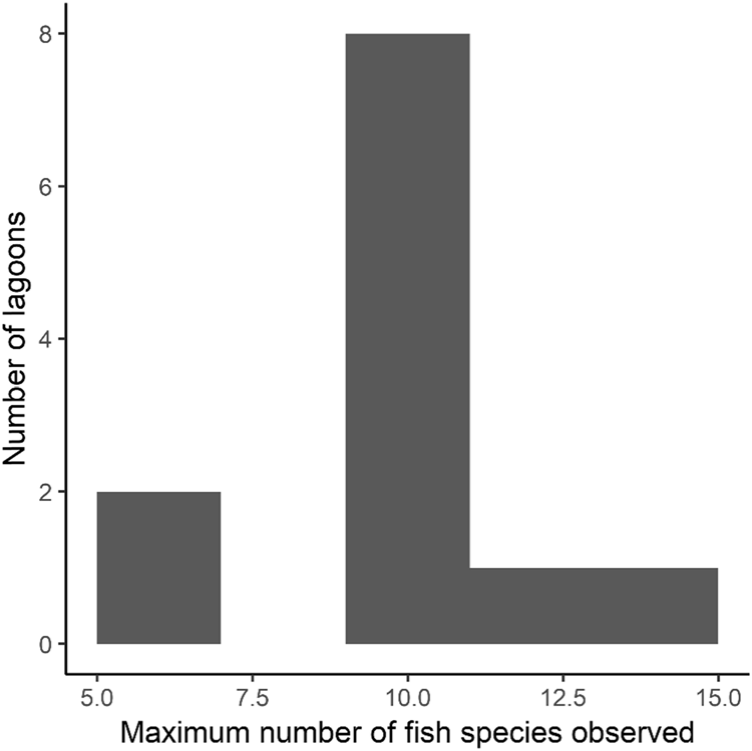
The maximum number of fish observed in each of the 11 SIIP-constructed lagoons under the Riversdale-Murray Scheme, surveyed using boat electric fishing in 2019 and 2020

Across all survey events, 36 native fishes and 4 non-native fishes were observed (Table 2). The mean species richness observed at each survey was 8.2 (range = 5–15; Fig. 2), while each survey caught, on average, 222 individuals (range = 35–838).

**Table 2:** Fish observed in scheme-funded wetlands surveyed in 2019 and 2020 using electric fishing

Family	Species	Common Name
Native species		
Ambassidae	*Ambassis agrammus*	Sailfin glass perch
Anguillidae	*Anguilla reinhardtii* ^§^	Speckled longfin eel
	*Anguilla obscura* ^§^	Pacific short-finned eel
Apogonidae	*Glossamia aprion*	Mouth Almighty
Ariidae	*Neoarius graeffei*	Lesser salmon catfish
Atherinidae	*Craterocephalus stercusmuscarum*	Fly speckled hardyhead
Belonidae	*Strongylura krefftii*	Freshwater longtom
Centropomidae	*Lates calcarifer §*	Barramundi
Chanidae	*Chanos chanos §*	Milkfish
Clupeidae	*Nematalosa erebi*	Bony bream
Eleotridae	*Giuris margaritacea §*	Snakehead gudgeon
	*Hypseleotris compressa §*	Empire gudgeon
	*Hypseleotris klunzingeri*	Western carp gudgeon
	*Hypseleotris galii*	Firetail gudgeon
	*Hypseleotris spp. 1*	Midgeley’s carp gudgeon
	*Mogurnda adspersa*	Purple-spotted gudgeon
	*Oxyeleotris lineolatus*	Sleepy cod
Elopidae	*Elops hawaiensis §*	Giant Herring
Engraulidae	*Thryssa scratchleyi §*	Freshwater anchovy
Gobiidae	*Redigobius bikolanus §*	Speckled goby
Kuhliidae	*Kuhlia rupestris §*	Jungle perch
Lutjanidae	*Lutjanus argentimaculatus §*	Mangrove jack
Megalopidae	*Megalops cyprinoides §*	Tarpon
Melanotaeniidae	*Melanotaenia splendida*	Eastern rainbow fish
Scatophagidae	*Scatophagus argus §*	Spotted scat
	*Selenotoca multifasciata §*	Banded scat
Synbranchidae	*Ophisternon gutturale*	Swamp eel
Terapontidae	*Amniataba percoides*	Barred grunter
	*Hephaestus fuliginosus*	Sooty grunter
	*Leiopotherapon* *unicolour*	Spangled perch
Toxotidae	*Toxotes chatareus*	Seven-spot Archerfish
	*Neosilurus ater*	Black catfish
	*Neosilurus hyrtlii*	Hyrtl’s tandan
	*Arrhampus sclerolepis*	Snub-nosed garfish
	*Porochilus rendahli*	Rendahl’s catfish
	*Scortum parviceps*	Small-headed grunter
Non-native fish species		
Cichlidae	*Oreochromis mossambicus*	Mozambique Tilapia
	*Tilapia mariae*	Spotted Tilapia
Osphronemidae	*Trichopodus trichopterus*	Three-spot gourami
Poeciliidae	*Gambusia holbrooki*	Eastern mosquitofish
		Total native species: 36

Most water quality samples exceeded the total nitrogen, ammonia and chlorophyll-a objectives, and all samples exceeded the total phosphorus objective (Table 3; Table S1). While the total dissolved nitrogen objective was largely met (small exceedances) and the total dissolved phosphorus objective was always met. All toxicants (pesticides and herbicides) tested were well within set objectives or guideline trigger values (Table S2), except for one sample at one site which exceeded the diuron trigger value (Note: the ANZECC (2000) disclaims the diuron trigger value as having high uncertainty). Of the 20 diurnal oxygen cycles assessed, all but three events had dissolved oxygen saturations that exceeded the default acute trigger value guideline of 30% (Tables S3 & S4; Butler and Burrows 2007).

**Table 3:** Summary statistics for water quality parameters measured at 11 SIIP constructed lagoons as grab samples in 2019 and 2020

	Total suspended sediments (mg/L)	Turbidity (NTU)	Total Nitrogen (µg N/L)	Total Dissolved Nitrogen (µg N/L)	Ammonia (µg N/L)	Total Phosphorus (µg P/L)	Total Dissolved Phosphorus (µg P/L)	Chlorophyll-a (µg/L)
Minimum	2	1.2	273	67	3	11	3	2.05
Median	9.85	11	418	260.5	14	23.5	8	7.465
Maximum	59	80	868	656	91	67	17	43.72

### Landholder Financial Assessment

#### Changes in Farm Practice

During the semi-structured interviews (*N* = 5, Note S1), cane farmers reported two forms of productivity improvement following elevation of fields adjacent to the drainage lagoon with excavated spoil. Two farmers reported that they had been able to grow cane on these areas prior to elevating them with excavated spoil. However, the typical cane production cycle for these areas prior to elevation comprised two ratoon harvests in addition to the plant cane harvest. Following elevation, this extended to four or five ratoon harvests following the plant cane harvest. A further three farmers reported that they had been unable to grow cane adjacent to the lagoon site before these areas were elevated, previously using them for cattle fattening. After elevation, these areas could support cane production with a cane cycle covering four ratoons following plant cane.

#### Construction and Maintenance Costs

Using the regression from Eq. (1) (R^2^ = 0.94, N = 44, Table S6), the total construction cost of a representative 0.3 ha lagoon (depth 3.5 m, excavated volume 10,500 m^3^) was estimated to be $20,053 in 2003 AUD, (equivalent to $29,900 in 2019 AUD$; Table 1). After the Riversdale-Murray Scheme paid 67% of lagoon construction cost, a representative farmer would have contributed approximately $6684 in 2003 AUD ($9967 in 2019 AUD$) towards this cost as a combination of in-kind and cash.

Farmers indicated, via the semi-structured interviews (*N* = 5), that annual maintenance of the drainage lagoon was largely limited to one annual application of glyphosate herbicide to control aquatic weeds, particularly the introduced invasive species *Hymenachne amplexicaulis* and hybrids. Treatment took 2–5 h annually and typically cost between $100 and $1000 (in 2020 AUD$ at the time of the interviews; equivalent to $99 to $990 in 2019 AUD$). Based on this information, we assumed a mid-range annual maintenance cost of $545 (in 2019 AUD$) for a representative lagoon. Other one-off maintenance actions, un-costed here, include maintaining steep edges and planting trees to reduce grass and *Hymenachne* growth and minor sediment removal.

#### Drainage

**Fig. 3:**
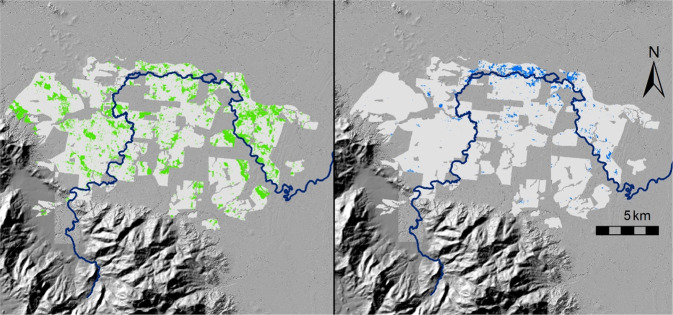
Spatial plots of 25 m × 25 m grid cells from Digital Earth Australia’s ‘Water observations from space’ dataset for which the integer-rounded percentage of ‘wet’ observations (**a**) decreased [green] and (**b**) increased [blue] after implementation of the Riversdale-Murray Valley Drainage Scheme. Cane land in the Scheme area is shaded light grey

Overall, the scheme resulted in greater water aggregation, with large areas becoming drier, while some areas became wetter (Fig. 3). Specifically, 1192 ha (i.e., 14.5% of cane land in the scheme area or 31,868 grid cells) recorded fewer wet observations, while 58 ha (i.e., 0.4% of cane area or 929 cells) showed increased inundation, after implementation of the Riversdale-Murray Valley Scheme (Fig. 3). There was no significant difference in average annual rainfall for the duration of the ‘Water observations from space’ datasets pre- and post-implementation of the drainage scheme (*p* = 0.244, Welch 2-sample *t* test; Fig. S1).

#### Benefit from Productivity Improvement on Elevated Land

Increasing from a two-ratoon to a four-ratoon production cycle across elevated land increased the estimated gross annual margin from $777/ha to $953/ha, an increase of $176/ha (all gross margins in 2019 AUD$; Tables 4 and 5). Roebeling et al. (2007) estimated that the gross margin from cattle fattening in the Tully-Murray catchment on poorly drained loam soils of alluvial origin could be maximized at a fertiliser application rate of 80 kgN/ha and a stocking density of 1.75 animal units/ha (where one animal unit equals 400 kg live weight). The resulting maximized annuity gross margin is $151/ha (in 2019 AUD$) (Roebeling et al. 2007; reported as $115/ha in 2007 AUD$). Converting from cattle fattening to 4-ratoon cane would, therefore, increase gross margins by $802/ha (in 2019 AUD$). Interviews with farmers indicated that land adjacent to the lagoon was typically elevated by 0.2 m–1.5 m when construction spoil was added. An elevation of 0.84 m is assumed this analysis (with 10,500 m^3^ of excavated spoil spread over 1.25 ha of land).

**Table 4:** Predicted gross margins under two and four-ratoon cane production system for a moderate permeability Mari soil in SILO climate zone 1800_14585 under Six Easy Steps fertiliser applications and legume fallow

	Plant cane + four ratoons with cow-pea legume fallow							Plant cane + two ratoons with cow-pea legume fallow				
	Late Plant	First Ratoon	Second Ratoon	Third Ratoon	Fourth Ratoon	Legume Fallow	Farm	Late Plant	First Ratoon	Second Ratoon	Legume Fallow	Farm
Area (ha)	25	25	25	25	25	25	150	37.5	37.5	37.5	37.5	150
Yield (tonne/ha) [cane only]	80.45	77.84	72.8	69.25	70.02	0	74.07	80.45	77.84	72.8	0	77.03
CCS (ha) [cane only]	13.08	12.73	12.55	12.33	12.64	0	12.68	13.08	12.73	12.55	0	12.79
Price ($/tonne) [cane only^a^]	38.15	36.70	35.95	35.04	36.33	0.00	36.43	38.15	36.70	35.95	0	36.93
Net Revenue ($/ha)	3069	2857	2618	2427	2544	0	2252	3069	2857	2618	0	2136
Growing costs ($/ha)	1605	545	545	545	545	635	736	1605	545	545	635	832
Harvesting costs ($/ha)	733	710	663	631	638	0	563	733	710	663	0	527
Variable costs ($/ha)	2338	1254	1208	1176	1182	635	1299	2338	1254	1208	635	1359
Gross Margin ($/ha)	731	1602	1410	1251	1361	−635	953	731	1602	1410	**−**635	777

Predictions produced by FEAT, using default parameter settings for the Tully Catchment (adjacent to the Murray) and yield predictions for SILO climate zone 1800_14585 from APSIM. Prices, costs, revenues and gross margins expressed in 2019 AUD$. (inflated from FEAT baseline predictions in 2015 AUD$ using ABS quarterly CPI-indices for Brisbane)

^a^Derived in FEAT using the industry standard cane pricing formula, a sugar price of $461, harvesting cost of $9.11/tonne, levies of $0.70/tonne (all in 2019 AUD$ escalated from the default 2015 AUD$ values in FEAT), and representative recoverable sugar content commonly called commercial cane sugar (CCS) percentages for each crop stage in the production cycle

**Table 5:** Return on investment from a representative farmer’s contribution to the construction and maintenance of a representative scheme-funded lagoon (Table 2) and soil spreading parcel (Table S5)

Costs (2019 AUD$)		
Lagoon construction (farmer contribution)	$9867	
Annual maintenance	$545	
Benefits (2019 AUD$)		
	Lower bound	Upper bound
Gross margin increment from improved drainage over 14% of farm area ($/year)	$984	$1967
*Productivity improvement from elevated area ($/year)*		
2-ratoons to 4-ratoons: gross margin increment ($/year)	$220	
Cattle fattening to 4-ratoons: gross margin increment ($/year)	$1003	
Financial performance (evaluated over a 15-year timeframe at a real discount rate of 5% p.a.)		
	Lower bound	Upper bound
*2 ratoons cane to 4 ratoons cane and improved drainage*		
Net present value (2019 AUD)	**−**3027	7182
Benefit cost ratio	0.81:1	1.46:1
Internal rate of return (%)	0.0	14.5
*Cattle fattening to 4 ratoons cane and improved drainage*		
Net present value (2019 AUD)	5093	15,302
Benefit cost ratio	1.33:1	1.99:1
Internal rate of return (%)	11.9	23.5

#### Net Financial Benefit

Reduced inundation from improved drainage across 14% of the area of a representative medium-sized farm was estimated to increase the annual gross margin by $984–1967 for the farm as a whole (in 2019 AUD$; Table 4). When the benefit from reduced inundation across 14% of the farm area is added to the benefit from converting from two-ratoon cane to four-ratoon cane on a representative 1.25 ha elevated soil parcel, less the costs that the farmer incurs in lagoon construction and maintenance, an internal rate of return ranging from 0.0 to 14.5% is achieved, with corresponding benefit:cost ratios of between 0.8:1 and 1.5:1 evaluated at a 5% discount rate over a 15-year timeframe. When the elevated land can be converted from cattle fattening to four-ratoon cane (in addition to the benefit from reduced inundation across the representative farm), the corresponding internal rate of return ranges from 11.9 to 23.5%, with corresponding benefit:cost ratios between 1.3:1 and 2.0:1 evaluated at a 5% discount rate over a 15-year timeframe (Table 5).

### Final Ecosystem Services

We identified 22 final ecosystem services, as per the Common International Classification of Ecosystem Services (Haines-Young and Potschin 2012), that are potentially supported by the Riversdale-Murray Scheme wetlands (Table 5). Of these, we have moderate or high confidence that nine of the benefits of these potential services are being realised. These benefits include: provision of wild animals (i.e., fish) for nutrition; water flow regulation; providing habitat for nursery populations and biodiversity; the promotion of both active and passive activities for recreation and mental health; supporting scientific inquiry; improving farm aesthetics; and having bequest value for future generations (Table 6).

**Table 6:** Final ecosystem services estimated to be provided by wetlands created as part of the Riversdale-Murray Scheme

Section	Class	Code	Application in Riversdale-Murray scheme	Ped.
Provisioning (Biotic)	Wild plants (terrestrial and aquatic, including fungi, algae) used for nutrition.	1.1.5.1	Indigenous community harvest (purpose unknown) observed by some farmers and relayed to us in person.	1
	Fibres and other materials from wild plants for direct use or processing (excluding genetic materials).	1.1.5.2	Indigenous community harvest (purpose unknown) observed by some farmers and relayed to us in person.	1
	Wild animals (terrestrial and aquatic) used for nutritional purposes.	1.1.6.1	Farmers and their family reported fishing in lagoons in interviews. Indigenous community harvest (purpose unknown) observed by some farmers and relayed to us in person and in interviews.	4
	Fibres and other materials from wild animals for direct use or processing (excluding genetic materials).	1.1.6.2	Indigenous community harvest (purpose unknown) observed by some farmers and relayed to us in person. Potential could include crocodile hide.	1
Regulation & Maintenance (Biotic)	Visual screening.	2.1.2.3	Farmers mentioned (in person) trees from wetland riparian screening unsightly land.	2
	Control of erosion rates.	2.2.1.1	Riparian vegetation may be reducing bank erosion.	1
	Hydrological cycle and water flow regulation (Including flood control, and coastal protection).	2.2.1.3	Observed change in farm inundation frequency, and hydrological modelling changes modelled and published by Karim et al (Karim et al. 2012).	4
	Wind protection.	2.2.1.4	Tall Eucalyptus trees were observed in in the riparian vegetation at some lagoon, and these may be protecting crops from wind, though yet to be quantified.	1
	Pollination (or ‘gamete’ dispersal in a marine context).	2.2.2.1	Wetland riparian vegetation may be supporting insect vectors that assist crop pollination. Not quantified.	1
	Maintaining nursery populations and habitats (Including gene pool protection).	2.2.2.3	Field surveys caught Barramundi across many wetlands of various sizes, indicating habitat support (Godfrey et al. 2016).	3
	Regulation of the chemical condition of freshwaters by living processes.	2.2.5.1	The wetlands likely provide some level of nutrient and sediment removal (Land et al. 2016; Adame et al. 2019b; Wallace and Waltham 2021).	2
	Regulation of chemical composition of atmosphere and oceans.	2.2.6.1	The wetlands will likely store carbon in deposited sediments and riparian vegetation. This is not quantified.	1
	Regulation of temperature and humidity, including ventilation and transpiration.	2.2.6.2	Open water and forested vegetation typically have a much lower albedo than crops and soil, which is likely to reduce ambient temperature. Wetlands can also increase humidity as retained water evaporates. The specific effect of this from these wetlands has not been quantified.	1
Cultural (Biotic)	Characteristics of living systems that that enable activities promoting health, recuperation or enjoyment through active or immersive interactions.	3.1.1.1	Multiple farmers have mentioned using the wetlands for fishing, kayaking, boating, skiing and walking in person and in interviews.	3
	Characteristics of living systems that enable activities promoting health, recuperation or enjoyment through passive or observational interactions.	3.1.1.2	Multiple farmers have mentioned visiting wetlands to enjoy the nature and to relax. They also mentioned personal satisfaction from completing the restoration.	3
	Characteristics of living systems that enable scientific investigation or the creation of traditional ecological knowledge.	3.1.2.1	Several scientific papers have arisen from examining these wetlands (Karim et al. 2012; Pearson et al. 2013; Godfrey et al. 2016).	4
	Characteristics of living systems that enable education and training.	3.1.2.2	Farmers have gained considerable knowledge from restoring and observing these wetlands.	2
	Characteristics of living systems that are resonant in terms of culture or heritage.	3.1.2.3	Indigenous people have been observed collecting from the lagoons, and this may resonate with their heritage/culture.	1
	Characteristics of living systems that enable aesthetic experiences.	3.1.2.4	Numerous farmers have mentioned in person and in interviews the pleasure they get from wetlands improving farm aesthetics.	3
	Characteristics or features of living systems that have an existence value.	3.2.2.1	The wetlands support a diverse array of freshwater fish, as observed here and in Godfrey et al. (2016).	3
	Characteristics or features of living systems that have an option or bequest value.	3.2.2.2	Farmers mentioned during visits and in interviews the satisfaction they get from providing a resource for their grandchildren to enjoy in the future.	3
Provisioning (Abiotic)	Surface water used as a material (non-drinking purposes).	4.2.1.2	Some farmers have used stored water for irrigation. The extent to which this occurs has not been quantified.	1

Class and codes are from the Common International Classification of Ecosystem Services (Haines-Young and Potschin 2012). Pedigree (Ped.) scores indicate confidence in service provision, ranging from 1 (low confidence) to 4 (total confidence), in line with those proposed by Costanza et al (Costanza et al. 1992)

## Discussion

Providing for the needs of agriculture and biodiversity in the same landscape is a significant challenge the world over, with heated debate over approaches for balancing competing demands. Here we observed that the integration of constructed lagoons throughout an intensive sugarcane dominated catchment in north Queensland to reducing flooding not only improved sugarcane profitability but provided habitat for freshwater biodiversity and potentially provide numerous other ecosystem services.

We found that the constructed lagoons provided sufficient habitat, water quality and connectivity to support high native fish diversity (Pearson et al. 2013; Karim et al. 2014), including commercially valuable species such as mangrove jack (*Lutjanus argentimaculatus*) and barramundi (*Lates calcarifer*), and iconic species such as the saltwater crocodile (*Crocodylus porosus*). Mangrove jack, for example, spawn near the outer reef and continental shelf, then migrate as juveniles to the shoreline, inhabiting mangrove roots, snags and rocks, gradually moving upriver and into lagoons as they mature. Once mature, after 2–11 years, adults begin to migrate back to spawning areas where they may reside for up to 40 years (Waltham et al. 2019). Barramundi, a diadromous fish, will ingress into coastal freshwater wetlands during wet season floods, to access important nursery habitat and forage in wetlands. As the lagoons were designed to be close to, and well connected to, the mainstem of the Tully River (Karim et al. 2014), migratory species are able to access and use the created habitat. The lagoons were also designed to be steep-sided and at least 1 m deep, which has helped to reduce macrophyte weed growth and provide habitable dissolved oxygen concentrations (Butler and Burrows 2007). As nutrient and phytoplankton (indicated by chlorophyl *a*) concentrations were high, reducing nutrient runoff into the lagoons may further improve the habitable condition and food web (Dodds and Smith 2016). Despite being artificial and draining intensive agricultural land, the wetlands support freshwater biodiversity and may provide alternative habitat to that lost from the draining of natural wetlands (Canning and Waltham 2021). Further work is required to examine the contribution of the lagoons towards providing habitat and food for aquatic birds and insects and delineating the habitats that support the greatest diversity for these groups.

In addition to the lagoons (and associated drains) supporting freshwater biodiversity, we estimated that they also improved on-farm profitability (from a landholder’s perspective) in two ways: (1) increased cane yield from improved drainage and flow regulation across a portion of the farm, and (2) increased cane yield from land that was elevated using wetland excavation spoil. Improved drainage and flow regulation reduced cane yield loss from waterlogging. Elevation of land with excavation spoil permitted two potential land use change scenarios to arise. The first was the conversion of cattle fattening land to production of sugarcane on a plant cane plus four ratoons cycle. The second was extension of the existing sugarcane production cycle from plant cane plus two ratoons to plant cane plus four ratoons.

It remains unexamined whether the scheme resulted in a net increase or decrease in nitrogen loading to the downstream Great Barrier Reef (GBR). An expansion and intensification of cane farming, along with increased drainage, may result in greater nitrogen leaching and runoff (Thorburn et al. 2011; Fraser et al. 2017). However, the constructed lagoon has the potential to denitrify runoff and offset any increased nitrogen losses (Land et al. 2016; Adame et al. 2019b; Wallace and Waltham 2021), while the increased cane growth, reduced tillage from longer cane cycles (increased ratooning) and reduced fertiliser loss (from flooding) may contribute to lower nitrogen losses (Webster et al. 2012; Skocaj et al. 2013; Thorburn et al. 2017). As the Great Barrier Reef requires substantial reductions in nitrogen loading to improve its ecological health (Brodie et al. 2012; Kroon et al. 2012; Wooldridge et al. 2015), if the approach resulted in a net increase in nitrogen loading to the reef, then decision-makers would need to consider a values trade-off. The health of the Great Barrier Reef, and the associated economic benefits from tourism and fisheries, would be pitted against improved sugarcane profitability and freshwater biodiversity. A net nitrogen reduction, however, would benefit the reef and could help attract funding that improves financial viability.

The extent to which nitrogen is removed by wetlands depends on many factors, including: the concentration and speciation nitrogen inflows; hydraulic loading rate, residence time and efficiency; temperature; wetland size and shape; composition of the ecological community (particularly the vegetation type and density); sediment type and composition; and oxygen concentrations and redox potential (Land et al. 2016; Alldred and Baines 2016; Vymazal 2017). Denitrification occurs in anaerobic conditions (negative oxidation-reduction (redox) potential) and when nitrate is used by denitrifying bacteria in respiration. Denitrifying bacteria are those with either the nirS, nirK, and nosZ genes, and use oxidised nitrogen compounds as a terminal electron acceptor in the absence of oxygen. Complete denitrification occurs in optimal conditions and released nitrogen gas as a by-product, whereas sub-optimal conditions lead to incomplete denitrification that releases N_2_O (a potent greenhouse gas) or NO_2_ as by-products (Burgin and Hamilton 2007; Martínez-Espinosa et al. 2021; Pinto et al. 2021). Complete denitrification is more probable when C:N ratios are high (e.g., >15–20) and anoxic conditions are persistent, if carbon becomes scarce or the environment becomes oxygenated then incomplete denitrification can occur (Klemedtsson et al. 2005). It is, therefore, essential that constructed wetlands with the goal of reducing nitrogen runoff are designed to have high hydrological residence time, persistent anoxic conditions and high carbon supply if it is to have low nitrous oxide emissions (Land et al. 2016; Oertel et al. 2016; Jahangir et al. 2016; Maucieri et al. 2017). Anoxic conditions can arise when soils are water-logged with minimal mixing, have minimal disturbance (such as mechanical ploughing and animal grazing) (Drewry et al. 2008), and when plant oxygenation rates are low as aquatic plants often oxygenate the soils proximal to their roots via aerenchyma transport (Oertel et al. 2016; Jahangir et al. 2016; Maucieri et al. 2017). Root oxygenation rates differ with functional guilds, growth stage, root density and depth, and the abundance of aerenchyma in tissue (Sorrell and Brix 2013; Alldred and Baines 2016). Given that the Tully-Murray catchment has substantial nitrogen runoff to the GBR, further research is recommended to evaluate the efficacy of the constructed wetlands in denitrifying runoff and whether improved design and management of the lagoons could lead to greater nutrient removal or retention. Given that denitrification requires systems to be anoxic, designing wetlands to support this function would require a values trade-off as anoxic conditions are not conducive to supporting biodiversity. It may be more appropriate for any future PES schemes to incentivise wetlands for denitrification in some instances and incentivise wetlands for biodiversity in other instances (Canning et al. 2021).

While these lagoons were profitable from a landholder’s perspective when a farm-wide benefit from reduced inundation and the Scheme’s 67% subsidy were included alongside the benefits from improved productivity on the elevated land adjacent to the lagoon, future schemes seeking to create wetlands in a similar way will also likely need to subsidize works. For lagoon creation to be profitable for a landholder, payments would likely need to ensure farmers achieve a benefit to cost ratio of at least 1:1 over the evaluation period (15 years in this study). To achieve a benefit to cost ratio of 1:1 over 15 years without the subsidy for lagoon construction and without including benefits from reduced inundation, but still accounting for the improved productivity on land elevated with excavated spoil, payments would need to be $8076/ha of wetland (in 2019 AUD$) when elevated land can be converted from cattle fattening to four-ratoon cane rotation, or $10,684/ha of wetland (in 2019 AUD$) when elevated land is converted from two-ratoon to four-ratoon cane rotation. Achieving a benefit to cost ratio of 1:1 over a shorter timeframe would be advantageous. It may be possible for future schemes to cover these costs by securing payments for ecosystem services, particularly if the payment scheme has sufficient flexibility to support multiple services, including those benefits that are non-rival and non-excludable (Canning et al. 2021). However, the annual payment rates required to achieve a benefit to cost ratio of 1:1 over a 15-year evaluation timeframe are much higher than the annual gross margins achieved from the land uses that preceded wetland conversion (by a factor of 14 with 2 ratoon cane as the prior land use, or by a factor of 54 with cattle fattening as the prior land use).

While we anticipate the lagoons providing, with various levels of confidence, at least 22 final ecosystem services (Haines-Young and Potschin 2012), not all services provide easily quantifiable benefits to clearly identifiable beneficiaries. Examples include the removal of nutrients and sediment, improvements to physical and mental health, and harvests from transient fisheries. Challenges in quantifying benefits and clearly attributing beneficiaries can make it difficult for these services to be recognized and rewarded through market-like schemes (Costanza et al. 2021). Having a scheme, that funds investment into wetland creation/restoration that does not rely heavily on benefit quantification, can accommodate multiple, bundled ecosystem services (including non-excludable and non-rival services), and is viable long-term to support ongoing maintenance, such as that facilitated by a common asset trust, may be the best option going forward (Canning et al. 2021; Costanza et al. 2021).

The results obtained here indicate a significant need to consider the retention and restoration of well-connected wetlands within the sustainable development of sugarcane landscapes. Wetlands have substantial ability to improve agricultural flood resilience, while providing wildlife habitat and ecosystem services. Recently, Saunders et al. (2022) identified barriers to the uptake of nature-based solutions, such as wetlands restoration, within Australia, along with key actions to address barriers. Recommendations included: (1) developing fit-for-purpose permitting processes for ecological restoration; (2) improving integrated mapping and classification of coastal ecosystems; (3) conducting research into the effective and risks of using restoration as nature-based solutions; (4) developing national-scale restoration guidelines that can cascade to state and local levels, including guidelines to support climate-resilient restorations; (5) develop decision-support models to help inform which actions to take under what circumstances; and (6) adapt the Restoration Opportunities Assessment Methodology (ROAM) to inform a systematic approach towards prioritization of restoration (Saunders et al. 2022). With respect to restoring wetlands for flood control, further work would be required to identify and prioritize locations where wetlands could be restored and yield a positive return on investment. If returns included accounting for other ecosystem services, then there may also be opportunities for funding from payment for ecosystem service schemes (Canning et al. 2021).

While this study demonstrates the potential benefits of an integrated catchment-scale wetlands restoration scheme, benefits may not be readily transferrable to other catchments without further assessment. Future schemes should use catchment-scale multi-property hydrological modelling to determine the ideal wetland sizes and positions on low-value agricultural land for regulating flood flows. Multi-criteria analysis could then be used to inform site selection by weighting locations and sizes that support the provision of other ecosystem services, such as carbon sequestration, nitrogen removal and supporting biodiversity.

In addition to using hydrological modelling to inform wetland design and connectivity for flood regulation, consideration should also be given to the hydrological connectivity needs of native fish assemblages. Across the studied lagoons, the temporal variation in fish assemblages is significantly influenced by lagoon connectivity with the downstream river, distance from the coast and flood dynamics (Karim et al. 2014; Arthington et al. 2015; Godfrey et al. 2016). Future schemes, particularly those in the Wet Tropics region, should still ensure there is a maintenance of seasonal patterns of flow and connectivity. Furthermore, wetlands should be designed to prevent the growth of exotic ponded-pasture grasses (such as *Hymenachne* and *Brachiaria mutica*) as these habitats supported the lowest fish species richness compared with other lagoon habitats (Arthington et al. 2015). This would include ensuring depth is greater than the tolerance of ponded-pasture grasses, riparian zones are well shaded with vegetation, and frequent flushing flows are maintained.

## Conclusion

Damage from flooding is a major risk for crop production, particularly in locations where agricultural land is established on previously drained wetland with high rainfall, and where rainfall is expected to become more extreme with climate change. Nature-based solutions, such as wetlands restoration, provide an alternative to conventional flood protection, such as establishing levee banks, as an avenue for achieving reduced flooding risk while supporting other values, such as biodiversity. In the present study, we demonstrate how the use of 44 lagoons and excavated silt traps over 16 properties substantially reduced crop flooding across Australia’s wettest catchment. Reduced flooding increased landholder gross margins for sugarcane cropping by allowing for a longer sugarcane cycle and the conversion of low-intensity cattle grazing areas into sugarcane. Further, we observed the wetlands providing for biodiversity by supporting 36 native fish species, along with the potential provision of 22 final ecosystem services. By rewarding the provision of ecosystem services in PES schemes, we show how the strategic restoration of wetlands for catchment-scale flood control can be a profitable nature-based solution. Those seeking similar benefits in other locations should make use of hydrological modelling to appropriately size and position wetlands across the landscape to achieve flood reduction goals and provide for the connectivity and flood regime needs of fish fauna. Further work is needed to quantify other benefits, such as carbon sequestration and water quality improvement, are recommended as they may support the development of viable supplemental income pathways under emerging PES schemes.

## Supplementary information

Supplementary informations

## References

[CR1] Acreman MC, Adams B, Birchall P, Connorton B (2000). Does groundwater abstraction cause degradation of rivers and wetlands?. Water Environ J.

[CR2] Adame M, Arthington A, Waltham N (2019). Managing threats and restoring wetlands within catchments of the Great Barrier Reef, Australia. Aquat Conserv.

[CR3] Adame M, Franklin H, Waltham N (2019). Nitrogen removal by tropical floodplain wetlands through denitrification. Mar Freshw Res.

[CR4] Alldred M, Baines SB (2016). Effects of wetland plants on denitrification rates: a meta-analysis. Ecol Appl.

[CR5] Allen GR, Midgley S, Allen M (2002). Field guide ot the freshwater fishes of Australia.

[CR6] Alluvium (2019) Effective and efficient pathways for investment in improved water quality in the Great Barrier Reef: Final report. A report for the Great Barrier Reef Foundation, Brisbane, Australia.

[CR7] American Public Health Association (APHA), American Water Works Association (AWWA), Water Environment Federation (WEF), et al. (2005) Standard Methods for the Examination of Water and Wastewater (21st Edition). Washington DC

[CR8] ANZECC (2000) Australian and New Zealand guidelines for fresh and marine water quality. Australian and New Zealand Environment and Conservation Council and Agriculture and Resource Management Council of Australia and New Zealand, Canberra 1–103

[CR9] Arthington A, Godfrey P, Pearson R (2015). Biodiversity values of remnant freshwater floodplain lagoons in agricultural catchments: evidence for fish of the Wet Tropics bioregion, northern Australia. Aquat Conserv.

[CR10] Australian Bureau of Statistics (2020) Consumer Price Index, Australia: 2020

[CR11] Awuchi CG, Awuchi CG, Ukpe AE (2020). Environmental impacts of food and agricultural production: a systematic review. Eur Acad Res.

[CR12] Bainbridge ZT, Brodie J, Faithful J (2009). Identifying the land-based sources of suspended sediments, nutrients and pesticides discharged to the Great Barrier Reef from the Tully–Murray Basin, Queensland, Australia. Mar Freshw Res.

[CR13] Banerjee S, Secchi S, Fargione J (2013). How to sell ecosystem services: a guide for designing new markets. Front Ecol Environ.

[CR14] Barbier EB (2019) Chapter 27 - The Value of Coastal Wetland Ecosystem Services. In: Perillo GME, Wolanski E, Cahoon DR, Hopkinson CSBT-CW (eds). Elsevier, pp 947–964

[CR15] Boyd J, Banzhaf S (2007). What are ecosystem services? The need for standardized environmental accounting units. Ecol Econ.

[CR16] Brander L, Brouwer R, Wagtendonk A (2013). Economic valuation of regulating services provided by wetlands in agricultural landscapes: A meta-analysis. Ecol Eng.

[CR17] Brodie J, Kroon F, Schaffelke B (2012). Terrestrial pollutant runoff to the Great Barrier Reef: an update of issues, priorities and management responses. Mar Pollut Bull.

[CR18] Brodie J, Waterhouse J (2012). A critical review of environmental management of the ‘not so Great’ Barrier Reef. Estuar Coast Shelf Sci.

[CR19] Buck J, Scheessele E, Relyea R, Blaustein A (2012). The effects of multiple stressors on wetland communities: pesticides, pathogens and competing amphibians. Freshw Biol.

[CR20] Burgin AJ, Hamilton SK (2007). Have we overemphasized the role of denitrification in aquatic ecosystems? A review of nitrate removal pathways. Front Ecol Environ.

[CR21] Butler B, Burrows DW (2007). Dissolved oxygen guidelines for freshwater habitats of northern Australia.

[CR22] Canegrowers (2020). Nitrogen management in the Queensland sugarcane industry.

[CR23] Canegrowers (2020). Nitrogen management in the Queensland sugarcane industry.

[CR24] Canning AD, Waltham NJ (2021). Ecological impact assessment of climate change and habitat loss on wetland vertebrate assemblages of the Great Barrier Reef catchment and the influence of survey bias.. Ecol Evol.

[CR25] Canning AD, Jarvis D, Costanza R (2021). Financial incentives for large-scale wetland restoration: beyond markets to common asset trusts. One Earth.

[CR26] Chen RZ, Wong M-H (2016). Integrated wetlands for food production. Environ Res.

[CR27] Coleman J, Huh O, Braud D (2008). Wetland loss in world deltas.. J Coast Res.

[CR28] Costanza R, Atkins P, Hernandez-Blanco M, Kubiszewski I (2021). Common asset trusts to effectively steward natural capital and ecosystem services at multiple scales. J Environ Manag.

[CR29] Costanza R, Funtowicz S, Ravetz J (1992). Assessing and communicating data quality in policy-relevant research. Environ Manag.

[CR30] Creighton C, Hobday AJ, Lockwood M, Pecl GT (2016). Adapting management of marine environments to a changing climate: a checklist to guide reform and assess progress. Ecosystems.

[CR31] Cunningham AB, Finlayson CM, Horwitz P, Weinstein P (2015). Wetlands and People’s Wellbeing: Basic Needs. Food Security and Medicinal Properties BT - Wetlands and Human Health.

[CR32] Davidson NC (2014). How much wetland has the world lost? Long-term and recent trends in global wetland area. Mar Freshw Res.

[CR33] Davidson NC, van Dam AA, Finlayson CM, McInnes RJ (2019). Worth of wetlands: revised global monetary values of coastal and inland wetland ecosystem services. Mar Freshw Res.

[CR34] Department of Environment and Science (2019) Addendum to Wetland Mapping and Classification Methodology - overall framework - A method to provide baseline mapping and classification for wetlands in Queensland. Brisbane, Australia.

[CR35] Department of Environment and Science (2018) Monitoring and Sampling Manual: Environmental Protection (Water) Policy. Brisbane.

[CR36] DERM (2009) Monitoring and Sampling Manual 2009.

[CR37] Dodds W, Smith V (2016). Nitrogen, phosphorus, and eutrophication in streams. Inland Waters.

[CR38] Drewry JJ, Cameron KC, Buchan GD (2008). Pasture yield and soil physical property responses to soil compaction from treading and grazinga review. Soil Res.

[CR39] Dudgeon D (2019). Multiple threats imperil freshwater biodiversity in the Anthropocene. Curr Biol.

[CR40] Environmental Protection Agency (2005) Wetland Mapping and Classification Methodology - A Method to Provide Baseline Mapping and Classification for Wetlands in Queensland, Version 1.2. Brisbane, Australia, Australia

[CR41] Ernst & Young (2001) Review of Sugar Industry Infrastructure Projects. Review by Ernst & Young for the Department of Agriculture, Fisheries and Forestry, Australia

[CR42] Finisdore J, Rhodes C, Haines-Young R, et al. (2020) The 18 benefits of using ecosystem services classification systems. Ecosyst Serv 45:. 10.1016/j.ecoser.2020.101160

[CR44] Fraser G, Rohde K, Silburn M (2017). Fertiliser management effects on dissolved inorganic nitrogen in runoff from Australian sugarcane farms. Environ Monit Assess.

[CR45] Gaugler T, Stoeckl S, Rathgeber AW (2020). Global climate impacts of agriculture: A meta-regression analysis of food production. J Clean Prod.

[CR46] Geoscience Australia Water observations from space. In: Digital Earth Australia: Water Observations from Space.

[CR47] Gliessman SR (2020). Transforming food and agriculture systems with agroecology. Agric Hum Values.

[CR48] Godfrey PC, Arthington AH, Pearson RG (2016). Fish larvae and recruitment patterns in floodplain lagoons of the Australian Wet Tropics. Mar Freshw Res.

[CR49] Haines-Young R, Potschin M (2012). Common international classification of ecosystem services (CICES, Version 4.1). Eur Environ Agency.

[CR50] Jahangir MMR, Richards KG, Healy MG (2016). Carbon and nitrogen dynamics and greenhouse gas emissions in constructed wetlands treating wastewater: A review. Hydrol Earth Syst Sci.

[CR51] Johnston RJ, Russell M (2011). An operational structure for clarity in ecosystem service values. Ecol Econ.

[CR52] Kadykalo AN, Findlay CS (2016). The flow regulation services of wetlands. Ecosyst Serv.

[CR53] Karim F, Kinsey-Henderson A, Wallace J (2012). Modelling wetland connectivity during overbank flooding in a tropical floodplain in north Queensland, Australia.. Hydrol Process.

[CR54] Karim F, Kinsey-Henderson A, Wallace J (2014). Modelling hydrological connectivity of tropical floodplain wetlands via a combined natural and artificial stream network.. Hydrol Process.

[CR57] Keating BA, Carberry PS, Hammer GL (2003). An overview of APSIM, a model designed for farming systems simulation. Eur J Agron.

[CR58] Klemedtsson L, Von Arnold K, Weslien P, Gundersen P (2005). Soil CN ratio as a scalar parameter to predict nitrous oxide emissions. Glob Chang Biol.

[CR59] Kroon FJ, Kuhnert PM, Henderson BL (2012). River loads of suspended solids, nitrogen, phosphorus and herbicides delivered to the Great Barrier Reef lagoon. Mar Pollut Bull.

[CR60] LaCanne CE, Lundgren JG (2018). Regenerative agriculture: Merging farming and natural resource conservation profitably.. PeerJ.

[CR61] Land M, Granéli W, Grimvall A (2016). How effective are created or restored freshwater wetlands for nitrogen and phosphorus removal? A systematic review. Environ Evid.

[CR63] Mace GM, Norris K, Fitter AH (2012). Biodiversity and ecosystem services: a multilayered relationship. Trends Ecol Evol.

[CR64] MacNeil MA, Mellin C, Matthews S (2019). Water quality mediates resilience on the Great Barrier Reef. Nat Ecol Evol.

[CR65] Martínez-Espinosa C, Sauvage S, Al Bitar A (2021). Denitrification in wetlands: A review towards a quantification at global scale. Sci Total Environ.

[CR66] Maucieri C, Barbera A, Vymazal J, Borin M (2017). A review on the main affecting factors of greenhouse gases emission in constructed wetlands. Agric Meteorol.

[CR67] McIntyre S, McGinness HM, Gaydon D, Arthur AD (2011). Introducing irrigation efficiencies: prospects for water-dependent biodiversity in a rice agro-ecosystem. Environ Conserv.

[CR68] Merrin M The formation of the Riversdale-Murray Valley Water Management Board: incorporating preliminary design for the Riversdale-Murray Valley Water Management Scheme. Final Report for the Local Management Group for the Riversdale-Murray Valley Infrastructure Project.

[CR69] Mueller N, Lewis A, Roberts D (2016). Water observations from space: Mapping surface water from 25years of Landsat imagery across Australia. Remote Sens Environ.

[CR70] Oertel C, Matschullat J, Zurba K (2016). Greenhouse gas emissions from soils—A review. Geochemistry.

[CR71] Ostrowski A, Connolly RM, Sievers M (2021). Evaluating multiple stressor research in coastal wetlands: A systematic review. Mar Environ Res.

[CR72] Pearson RG, Godfrey PC, Arthington AH (2013). Biophysical status of remnant freshwater floodplain lagoons in the Great Barrier Reef catchment: a challenge for assessment and monitoring. Mar Freshw Res.

[CR73] Peh KS-H, Balmford A, Field RH (2014). Benefits and costs of ecological restoration: Rapid assessment of changing ecosystem service values at a U.K. wetland. Ecol Evol.

[CR76] Pinto R, Weigelhofer G, Brito AG, Hein T (2021). Effects of dry-wet cycles on nitrous oxide emissions in freshwater sediments: a synthesis. PeerJ.

[CR77] Pusey BJ, Kennard MJ, Arthur JM, Arthington AH (1998). Quantitative sampling of stream fish assemblages: Single- vs multiple-pass electrofishing. Aust J Ecol.

[CR78] (2015) Land use mapping -1999 to 2015 - Wet TropicsNRM region. Queensland Government, Brisbane, Retrieved from http://qldspatial.information.qld.gov.au/catalogue/custom/viewMetadataDetails.page?uuid=%7B273F1E50-DD95-4772-BD6C-5C1963CAA594%7D.

[CR79] Ramsar Convention Secretariat (2016) An introduction to the Convention on Wetlands (previously The Ramsar Convention Manual). Gland, Switzerland

[CR80] Roebeling PC, Webster AJ, Biggs J, Thorburn P (2007). Financial-economic analysis of current best management practices for sugarcane, horticulture, grazing and forestry industries in the Tully-Murray catchment.

[CR81] Sapkota Y, White JRJR (2020). Carbon offset market methodologies applicable for coastal wetland restoration and conservation in the United States: A review. Sci Total Environ.

[CR82] Saunders M, Waltham N, Cannard T, et al. (2022) A roadmap for coordinated landscape-scale coastal and marine ecosystem restoration. Cairns, Australia

[CR83] Segan DB, Murray KA, Watson JEM (2016). A global assessment of current and future biodiversity vulnerability to habitat loss–climate change interactions. Glob Ecol Conserv.

[CR84] Shennan C, Bode CA (2002) Integrating wetland habitat with agriculture. The farm as a natural habitat (eds. LL Jackson & D Jackson) 189–204

[CR85] Skocaj DM, Everingham YL, Schroeder BL (2013). Nitrogen Management Guidelines for Sugarcane Production in Australia: Can These Be Modified for Wet Tropical Conditions Using Seasonal Climate Forecasting?. Springe Sci Rev.

[CR86] Sorrell BK, Brix H (2013). Gas transport and exchange through wetland plant aerenchyma. Methods Biogeochemistry Wetl.

[CR87] State of Queensland (2016) FEAT Regional Scenarios: Major parameter information sheet. Brisbane, Australia.

[CR88] State of Queensland Department of Agriculture and Fisheries (2020) FEAT - Farm Economic Analysis Tool.

[CR89] State of Queensland (Department of Resources) (2021) Queensland basemap topographic web service. https://spatial-gis.information.qld.gov.au/arcgis/rest/services/Basemaps/QldMap_Topo/MapServer/WMSServer? Accessed 16 Sep 2022

[CR90] Thorburn PJ, Biggs JS, Attard SJ, Kemei J (2011). Environmental impacts of irrigated sugarcane production: Nitrogen lost through runoff and leaching. Agric Ecosyst Environ.

[CR91] Thorburn PJ, Biggs JS, Palmer J (2017). Prioritizing crop management to increase nitrogen use efficiency in australian sugarcane crops. Front Plant Sci.

[CR92] Tilman D, Cassman KG, Matson PA (2002). Agricultural sustainability and intensive production practices. Nature.

[CR93] Tilman D, Fargione J, Wolff B (2001). Forecasting agriculturally driven global environmental change. Science (1979).

[CR94] United Nations General Assembly (2015). Transforming our world: the 2030 Agenda for Sustainable Development.

[CR95] van Coppenolle R, Temmerman S (2019). A global exploration of tidal wetland creation for nature-based flood risk mitigation in coastal cities. Estuar Coast Shelf Sci.

[CR96] van Dijk M, Morley T, Rau ML, Saghai Y (2021). A meta-analysis of projected global food demand and population at risk of hunger for the period 2010–2050. Nat Food.

[CR97] Verhoeven JTA, Setter TL (2010). Agricultural use of wetlands: opportunities and limitations. Ann Bot.

[CR98] Vymazal J (2017). The use of constructed wetlands for nitrogen removal from agricultural drainage: A review.. Sci agriculturae Bohem.

[CR99] Wallace J, Waltham NJ (2021). On the potential for improving water quality entering the Great Barrier Reef lagoon using constructed wetlands. Mar Pollut Bull.

[CR100] Waltham N, Burrows D, Wegscheidl C (2019). Lost floodplain wetland environments and efforts to restore connectivity, habitat and water quality settings on the Great Barrier Reef. Front Mar Sci.

[CR101] Waltham N, Elliott M, Lee SY, et al. (2020) UN Decade on Ecosystem of Restoration 2021–2030—what chance for success in restoring coastal ecosytems? Front Mar Sci

[CR102] Waterhouse J, Brodie J, Lewis S, Audas D (2016). Land-sea connectivity, ecohydrology and holistic management of the Great Barrier Reef and its catchments: time for a change. Ecohydrol Hydrobiol.

[CR103] Webster AJ, Bartley R, Armour JD (2012). Reducing dissolved inorganic nitrogen in surface runoff water from sugarcane production systems. Mar Pollut Bull.

[CR104] Wong CP, Jiang B, Kinzig AP (2015). Linking ecosystem characteristics to final ecosystem services for public policy. Ecol Lett.

[CR105] Wooldridge SA, Brodie JE, Kroon FJ, Turner RDR (2015). Ecologically based targets for bioavailable (reactive) nitrogen discharge from the drainage basins of the Wet Tropics region, Great Barrier Reef.. Marine Pollution Bulletin.

[CR106] Xu X, Chen M, Yang G (2020). Wetland ecosystem services research: A critical review. Glob Ecol Conserv.

