# Supplementary material for: Constructed Wetlands Suitability for Sugarcane Profitability, Freshwater Biodiversity and Ecosystem Services
Source: Environ Manage. 2022 Oct 21;71(2):304–20. doi: 10.1007/s00267-022-01734-4 (PMC9892086; doi:10.1007/s00267-022-01734-4)
Supplement: Supplementary file 1 — Supplementary informations [file 267_2022_1734_MOESM1_ESM.docx]

Supplementary information for:

**Sugarcane profitability, freshwater biodiversity, and ecosystem services are all enhanced by constructed wetlands**

Adam D. Canning^1a^, James C.R. Smart^2^, Joshua Dyke^3^, Graeme Curwen^3^, Syezlin Hasan^3^, & Nathan J. Waltham^1^

^1^Centre for Tropical Water and Aquatic Ecosystem Research (TropWATER), James Cook University, 1 James Cook Drive, Townsville, 4811, Australia

^2^Australian Rivers Institute and School of Environment and Science, 170 Kessels Road, Nathan, Queensland 4111, Australia

^3^Australian Rivers Institute, Griffith University, 170 Kessels Road, Nathan, Queensland 4111, Australia

^a^Corresponding author: adam.canning@jcu.edu.au

| Table S1. Water quality concentrations from grab samples collected at each site in October 2019 and June 2020. TSS, Total Suspended Sediment; PN, particulate nitrogen; PP, particulate phosphorus. Relevant water quality (WQ) standards are those from the *Environmental Protection (Water and Wetland Biodiversity) Policy 2019: Tully River, Murray River and Hinchinbrook Island Basins Environmental Values and Water Quality Objectives*, Butler and Burrows (2007), or the ANZECC (2000) trigger values. | | | | | | | | | | | | | | | |
| --- | --- | --- | --- | --- | --- | --- | --- | --- | --- | --- | --- | --- | --- | --- | --- |
|  | Date | TSS (mg/L) | Turbidity  (NTU) | Total Nitrogen (µg N/L) | Total Dissolved Nitrogen (µg N/L) | Ammonia (µg N/L) | Nitrate-N (µg N/L) | Nitrite-N (µg N/L) | PN  (µg N/L) | Total Phosphorus (µg P/L) | Total Dissolved Phosphorus (µg P/L) | PP  (µg P/L) | Filterable Reactive Phosphorus (µg P/L) | Chlorophyll-*a* (µg/L) | Phaeophytin-*a* (µg/L) |
| WQ Standard | - | - | 200 | 350 | 350 | 10 | - | - | - | 10 | - | - | 5 | 3 | - |
| T1 | 6/10/2019 | 2 | 1.2 | 278 | 255 | 18 | 7 | 1 | 23 | 14 | 8 | 6 | 2 | 2.64 | 1.18 |
| T2 | 5/10/2019 | 6 | 5.6 | 552 | 362 | 20 | 4 | 1 | 190 | 30 | 8 | 22 | 3 | 7.44 | 1.32 |
| T3 | 2/10/2019 | 6.1 | 6.2 | 422 | 357 | 24 | 5 | 2 | 65 | 27 | 15 | 12 | 3 | 4.95 | 1.95 |
| T4 | 4/10/2019 | 15 | 13 | 344 | 237 | 17 | 4 | 1 | 107 | 24 | 8 | 16 | 3 | 9.85 | 0.92 |
| T5 | 2/10/2019 | 41 | 55 | 868 | 656 | 91 | 62 | 3 | 212 | 34 | 13 | 21 | 4 | 7.34 | 2.31 |
| T6 | 6/10/2019 | 19 | 16 | 592 | 313 | 8 | 4 | 1 | 279 | 44 | 11 | 33 | 4 | 8.12 | 2.04 |
| T7 | 6/10/2019 | 7.2 | 3.9 | 384 | 376 | 3 | 5 | 1 | 8 | 17 | 8 | 9 | 3 | 5.22 | 1.1 |
| T8 | 4/10/2019 | 4.1 | 2.5 | 273 | 209 | 20 | 2 | 2 | 64 | 17 | 7 | 10 | 3 | 5.16 | 1.65 |
| T9 | 3/10/2019 | 7.7 | 9.3 | 414 | 358 | 7 | 4 | 1 | 56 | 16 | 8 | 8 | 2 | 4.13 | 2.39 |
| T10 | 3/10/2019 | 18 | 12 | 446 | 160 | 10 | 2 | 2 | 286 | 23 | 8 | 15 | 3 | 8.86 | 2.65 |
| T11 | 3/10/2019 | 34 | 37 | 450 | 312 | 4 | 6 | 2 | 138 | 22 | 8 | 14 | 3 | 4.82 | 2.43 |
| T12 | 5/10/2019 | 15 | 16 | 468 | 286 | 12 | 4 | 1 | 182 | 32 | 7 | 25 | 3 | 7.79 | 1.52 |
| T1 | 27/06/2020 | 6.8 | 6.2 | 487 | 114 | 10 | 3 | 2 | 373 | 67 | 7 | 60 | 3 | 32.09 | 5.58 |
| T2 | 23/06/2020 | 5.6 | 5.4 | 298 | 196 | 31 | 47 | 3 | 102 | 20 | 12 | 8 | 6 | 7.49 | 2.23 |
| T3 | 24/06/2020 | 5.6 | 5.3 | 276 | 149 | 14 | 11 | 2 | 127 | 22 | 6 | 16 | 2 | 18.17 | 1.53 |
| T4 | 27/06/2020 | 4.2 | 5.4 | 349 | 270 | 31 | 124 | 2 | 79 | 11 | 3 | 8 | <1 | 2.05 | 0.84 |
| T5 | 26/06/2020 | 7.5 | 10 | 274 | 195 | 8 | 15 | 2 | 79 | 15 | 5 | 10 | <1 | 3.36 | 1.53 |
| T6 | 23/06/2020 | 12 | 12 | 480 | 266 | 9 | 168 | 4 | 214 | 54 | 17 | 37 | 3 | 43.72 | 1.89 |
| T9 | 25/06/2020 | 25 | 24 | 383 | 115 | 14 | 5 | 2 | 268 | 33 | 5 | 28 | 2 | 22.58 | 5.58 |
| T10 | 25/06/2020 | 14 | 12 | 507 | 211 | 5 | 82 | 4 | 296 | 42 | 7 | 35 | 3 | 34.86 | 4.79 |
| T11 | 25/06/2020 | 12 | 16 | 394 | 268 | 14 | 218 | 3 | 126 | 14 | 3 | 11 | 3 | 5.84 | 1.12 |
| T12 | 28/06/2020 | 59 | 80 | 540 | 67 | 14 | 5 | 2 | 473 | 64 | 5 | 59 | 3 | 10.97 | 3.16 |

Table S2. Toxicant concentrations from grab samples collected at each site in October 2019 and June 2020. Relevant water quality (WQ) standards are those from the ANZECC (2000) guidelines.

| Site | Date | Chlorpyrifos µg/L | Malathion µg/L | Diazinon µg/L | Pirimiphos-methyl µg/L | Thiobencarb µg/L | Pendimethalin µg/L | Hexazinone µg/L | Propiconazole µg/L | Hexaconazole µg/L | Difenoconazole µg/L | Tebuconazole µg/L | Flusilazole µg/L | Penconazole µg/L | Diuron µg/L | Ametryn µg/L | Atrazine µg/L | Cyanazine µg/L | Prometryn µg/L | Propazine µg/L | Simazine µg/L | Terbuthylazine µg/L | Terbutryn µg/L |
| --- | --- | --- | --- | --- | --- | --- | --- | --- | --- | --- | --- | --- | --- | --- | --- | --- | --- | --- | --- | --- | --- | --- | --- |
| WQ standard | - | 0.01 | 0.05 | 0.01 | - | 2.8 | - | 78 | - | - | - | - | - | - | 0.2 | - | 13 | - | - | - | 3.2 | - | - |
| T1 | 6/10/2019 | <0.001 | <0.001 | <0.0002 | <0.0002 | <0.0002 | <0.001 | 0.0311 | <0.0002 | <0.0002 | <0.0002 | <0.0002 | <0.0002 | <0.0002 | 0.0161 | <0.0002 | 0.0149 | <0.0002 | <0.0002 | <0.0002 | <0.0002 | <0.0002 | <0.0002 |
| T2 | 5/10/2019 | <0.001 | <0.001 | <0.0002 | <0.0002 | <0.0002 | <0.001 | 0.0079 | <0.0002 | <0.0002 | <0.0002 | <0.0002 | <0.0002 | <0.0002 | 0.0022 | 0.0008 | 0.0028 | <0.0002 | <0.0002 | <0.0002 | 0.0006 | <0.0002 | <0.0002 |
| T3 | 2/10/2019 | <0.001 | <0.001 | <0.0002 | <0.0002 | <0.0002 | <0.001 | 0.0312 | <0.0002 | <0.0002 | <0.0002 | <0.0002 | <0.0002 | <0.0002 | 0.0171 | <0.0002 | 0.0014 | <0.0002 | <0.0002 | <0.0002 | <0.0002 | <0.0002 | <0.0002 |
| T4 | 4/10/2019 | <0.001 | <0.001 | <0.0002 | <0.0002 | <0.0002 | <0.001 | 0.0136 | <0.0002 | <0.0002 | <0.0002 | <0.0002 | <0.0002 | <0.0002 | 0.0153 | 0.0003 | 0.0030 | <0.0002 | <0.0002 | <0.0002 | <0.0002 | <0.0002 | <0.0002 |
| T5 | 2/10/2019 | <0.001 | <0.001 | <0.0002 | <0.0002 | <0.0002 | <0.001 | 0.0060 | <0.0002 | <0.0002 | <0.0002 | <0.0002 | <0.0002 | <0.0002 | 0.0084 | 0.0003 | 0.0089 | <0.0002 | <0.0002 | <0.0002 | <0.0002 | <0.0002 | <0.0002 |
| T6 | 6/10/2019 | <0.001 | <0.001 | <0.0002 | <0.0002 | <0.0002 | <0.001 | 0.213 | <0.0002 | <0.0002 | <0.0002 | <0.0002 | <0.0002 | <0.0002 | 0.460 | 0.0015 | 0.0488 | <0.0002 | <0.0002 | 0.0003 | 0.0003 | <0.0002 | <0.0002 |
| T7 | 6/10/2019 | <0.001 | <0.001 | <0.0002 | <0.0002 | <0.0002 | <0.001 | 0.0080 | 0.0002 | <0.0002 | <0.0002 | <0.0002 | <0.0002 | <0.0002 | 0.0058 | 0.0002 | 0.0040 | <0.0002 | <0.0002 | <0.0002 | <0.0002 | <0.0002 | <0.0002 |
| T8 | 4/10/2019 | <0.001 | <0.001 | <0.0002 | <0.0002 | <0.0002 | <0.001 | 0.0392 | <0.0002 | <0.0002 | <0.0002 | <0.0002 | <0.0002 | <0.0002 | 0.0086 | 0.0002 | 0.0024 | <0.0002 | <0.0002 | <0.0002 | <0.0002 | <0.0002 | <0.0002 |
| T9 | 3/10/2019 | <0.001 | <0.001 | <0.0002 | <0.0002 | <0.0002 | <0.001 | 0.0424 | <0.0002 | <0.0002 | <0.0002 | <0.0002 | <0.0002 | <0.0002 | 0.0532 | 0.0012 | 0.0067 | <0.0002 | <0.0002 | <0.0002 | <0.0002 | <0.0002 | <0.0002 |
| T10 | 3/10/2019 | <0.001 | <0.001 | <0.0002 | <0.0002 | <0.0002 | <0.001 | 0.0636 | <0.0002 | <0.0002 | <0.0002 | <0.0002 | <0.0002 | <0.0002 | 0.0371 | 0.0010 | 0.0032 | <0.0002 | <0.0002 | <0.0002 | <0.0002 | <0.0002 | <0.0002 |
| T11 | 3/10/2019 | <0.001 | <0.001 | <0.0002 | <0.0002 | <0.0002 | <0.001 | 0.0764 | <0.0002 | <0.0002 | <0.0002 | <0.0002 | <0.0002 | <0.0002 | 0.0042 | 0.0013 | 0.0055 | <0.0002 | <0.0002 | <0.0002 | <0.0002 | <0.0002 | <0.0002 |
| T12 | 5/10/2019 | <0.001 | <0.001 | <0.0002 | <0.0002 | <0.0002 | <0.001 | 0.0062 | <0.0002 | <0.0002 | <0.0002 | <0.0002 | <0.0002 | <0.0002 | 0.0137 | 0.0005 | 0.158 | <0.0002 | <0.0002 | 0.0014 | 0.0005 | <0.0002 | <0.0002 |
| T1 | 27/06/2020 | <0.001 | <0.001 | <0.0002 | <0.0002 | <0.0002 | <0.001 | 0.0308 | <0.0002 | <0.0002 | <0.0002 | <0.0002 | <0.0002 | <0.0002 | <0.0200 | 0.0004 | 0.0292 | <0.0002 | <0.0002 | 0.0003 | <0.0002 | <0.0002 | <0.0002 |
| T2 | 23/06/2020 | <0.001 | <0.001 | <0.0002 | <0.0002 | <0.0002 | <0.001 | 0.0232 | <0.0002 | <0.0002 | <0.0002 | <0.0002 | <0.0002 | <0.0002 | 0.0288 | 0.0005 | <0.0200 | <0.0002 | 0.0004 | 0.0002 | <0.0200 | <0.0002 | <0.0002 |
| T3 | 24/06/2020 | <0.001 | <0.001 | <0.0002 | <0.0002 | <0.0002 | <0.001 | 0.0240 | <0.0002 | <0.0002 | <0.0002 | <0.0002 | <0.0002 | <0.0002 | <0.0200 | <0.0002 | <0.0200 | <0.0002 | 0.0006 | <0.0002 | <0.0002 | <0.0002 | <0.0002 |
| T4 | 27/06/2020 | <0.001 | <0.001 | <0.0002 | <0.0002 | <0.0002 | <0.001 | 0.0028 | <0.0002 | <0.0002 | <0.0002 | <0.0002 | <0.0002 | <0.0002 | 0.0038 | <0.0002 | <0.0020 | <0.0002 | 0.0006 | <0.0002 | <0.0002 | <0.0002 | <0.0002 |
| T5 | 26/06/2020 | <0.001 | <0.001 | <0.0002 | <0.0002 | <0.0002 | <0.001 | 0.0125 | <0.0002 | <0.0002 | <0.0002 | <0.0002 | <0.0002 | <0.0002 | 0.0035 | 0.0003 | 0.0020 | <0.0002 | 0.0002 | <0.0002 | 0.0003 | <0.0002 | <0.0002 |
| T6 | 23/06/2020 | <0.001 | <0.001 | <0.0002 | <0.0002 | <0.0002 | <0.001 | 0.0248 | <0.0002 | <0.0002 | <0.0002 | <0.0002 | <0.0002 | <0.0002 | <0.0200 | 0.0006 | <0.0200 | <0.0002 | 0.0010 | <0.0002 | <0.0002 | <0.0002 | <0.0002 |
| T9 | 25/06/2020 | <0.001 | <0.001 | <0.0002 | <0.0002 | <0.0002 | <0.001 | 0.0248 | <0.0002 | <0.0002 | <0.0002 | <0.0002 | <0.0002 | <0.0002 | 0.0440 | 0.0011 | <0.0200 | <0.0002 | 0.0006 | <0.0002 | <0.0002 | <0.0002 | <0.0002 |
| T10 | 25/06/2020 | <0.001 | <0.001 | <0.0002 | <0.0002 | <0.0002 | <0.001 | 0.0248 | <0.0002 | <0.0002 | <0.0002 | <0.0002 | <0.0002 | <0.0002 | 0.0079 | <0.0002 | <0.0020 | <0.0002 | 0.0002 | <0.0002 | <0.0002 | <0.0002 | <0.0002 |
| T11 | 25/06/2020 | <0.001 | <0.001 | <0.0002 | <0.0002 | <0.0002 | <0.001 | 0.0292 | <0.0002 | <0.0002 | <0.0002 | <0.0002 | <0.0002 | <0.0002 | 0.0084 | 0.0002 | 0.0030 | <0.0002 | 0.0007 | <0.0002 | <0.0002 | <0.0002 | <0.0002 |
| T12 | 28/06/2020 | <0.001 | <0.001 | <0.0002 | <0.0002 | <0.0002 | <0.001 | <0.0200 | <0.0002 | <0.0002 | <0.0002 | <0.0002 | <0.0002 | <0.0002 | 0.0024 | <0.0002 | 0.0015 | <0.0002 | <0.0002 | <0.0002 | 0.528 | <0.0002 | <0.0002 |

| **Table S3.** Depth profiling statistics for pH, electrical conductivity (EC), dissolved oxygen (DO) and temperature at 12 Riversdale-Murray Scheme lagoons in 2019 and 2020. N is the number of profile reads per survey. | | | | | | | | | | | |
| --- | --- | --- | --- | --- | --- | --- | --- | --- | --- | --- | --- |
| **Site** | **Date** | **Start Time** | **Max Reading Depth (m)** | **N** | **Min pH** | **Max pH** | **Mean EC (µS/cm)** | **Min DO (% sat)** | **Max DO (% sat)** | **Min Temp (ºC)** | **Max Temp (ºC)** |
| T3 | 02-Oct-19 | 14:20:00 | 3.50 | 19 | 6.02 | 7.53 | 46.0 | 10.8 | 92.8 | 23.8 | 28.9 |
| T1 | 06-Oct-19 | 9:10:00 | 3.50 | 29 | 5.87 | 6.27 | 40.0 | 2.0 | 31.8 | 24.5 | 25.9 |
| T4 | 02-Oct-19 | 10:30:00 | 2.25 | 24 | 6.31 | 7.29 | 63.1 | 17.8 | 80.0 | 25.2 | 27.6 |
| T7 | 06-Oct-19 | 15:30:00 | 1.70 | 19 | 5.94 | 6.59 | 48.5 | 0.8 | 82.9 | 25.8 | 28.9 |
| T9 | 03-Oct-19 | 9:30:00 | 1.75 | 23 | 6.72 | 7.20 | 49.1 | 69.6 | 80.8 | 26.8 | 27.0 |
| T10 | 03-Oct-19 | 11:00:00 | 1.50 | 20 | 6.26 | 7.01 | 52.8 | 16.8 | 68.3 | 24.6 | 26.9 |
| T11 | 03-Oct-19 | 13:10:00 | 1.50 | 21 | 6.43 | 7.28 | 53.5 | 35.1 | 84.3 | 25.7 | 29.6 |
| T2 | 05-Oct-19 | 15:15:00 | 4.00 | 27 | 6.25 | 6.72 | 48.6 | 38.2 | 83.6 | 25.8 | 27.9 |
| T8 | 04-Oct-19 | 15:50:00 | 5.00 | 27 | 6.58 | 7.38 | 52.5 | 5.1 | 90.3 | 25.1 | 27.7 |
| T4 | 04-Oct-19 | 11:00:00 | 2.00 | 17 | 6.10 | 7.15 | 56.6 | 4.6 | 75.9 | 25.4 | 29.2 |
| T12 | 05-Oct-19 | 11:30:00 | 0.75 | 11 | 6.09 | 6.83 | 62.4 | 1.9 | 93.0 | 23.6 | 27.8 |
| T6 | 06-Oct-19 | 12:30:00 | 1.35 | 18 | 6.49 | 7.02 | 74.6 | 36.5 | 99.5 | 25.7 | 29.0 |
| T1 | 06-Oct-19 | 9:10:00 | 3.50 | 26 | 5.87 | 6.27 | 40.0 | 2.0 | 29.1 | 24.5 | 26.0 |
| T7 | 06-Oct-19 | 15:30:00 | 1.70 | 19 | 5.94 | 6.59 | 48.6 | 0.8 | 82.9 | 25.9 | 28.9 |
| T6 | 23-Jun-20 | 10:40:00 | 2.10 | 25 | 6.45 | 7.03 | 76.7 | 0.9 | 103.0 | 21.4 | 24.4 |
| T2 | 23-Jun-20 | 14:15:00 | 4.50 | 27 | 5.95 | 7.03 | 42.4 | 1.3 | 70.7 | 23.1 | 25.8 |
| T3 | 24-Jun-20 | 11:00:00 | 3.50 | 26 | 5.83 | 6.55 | 47.0 | 26.0 | 58.6 | 20.3 | 22.4 |
| T11 | 25-Jun-20 | 11:05:00 | 2.30 | 24 | 6.11 | 6.97 | 61.0 | 3.7 | 78.1 | 21.1 | 22.7 |
| T9 | 25-Jun-20 | 13:10:00 | 1.90 | 25 | 6.21 | 7.35 | 50.5 | 8.6 | 97.1 | 22.5 | 24.9 |
| T10 | 25-Jun-20 | 15:00:00 | 1.50 | 19 | 6.22 | 7.54 | 59.3 | 23.0 | 98.0 | 21.3 | 23.0 |
| T5 | 26-Jun-20 | 15:30:00 | 3.50 | 27 | 5.60 | 6.30 | 56.6 | 2.8 | 97.1 | 21.6 | 24.7 |
| T4 | 27-Jun-20 | 11:20:00 | 2.75 | 20 | 5.41 | 6.35 | 40.5 | 30.4 | 52.7 | 20.1 | 21.9 |
| T1 | 27-Jun-20 | 14:00:00 | 5.10 | 26 | 5.45 | 6.41 | 37.0 | 2.5 | 51.9 | 20.4 | 23.2 |
| T12 | 28-Jun-20 | 14:40:00 | 0.75 | 4 | 5.67 | 5.88 | 42.3 | 38.8 | 43.9 | 21.5 | 22.6 |

| Table S4. Summary statistics for multi-parameter loggers in wetlands during the late dry season (October 2019) and post wet (June 2020) season surveys. Parameters are temperature (temp), electrical conductivity (EC), pH, and dissolved oxygen (DO). | | | | | | | | | | | | | | |
| --- | --- | --- | --- | --- | --- | --- | --- | --- | --- | --- | --- | --- | --- | --- |
| Site | Start Time | Finish Time | Min Temp | Max Temp | Mean Temp | Min EC | Max EC | Mean EC | Min pH | Max pH | Mean pH | Min DO | Max DO | Mean DO |
| T1 | 6/10/2019 9:40 | 7/10/2019 14:20 | 24.86 | 28.15 | 26.01 | 44 | 45 | 44 | 5.57 | 5.82 | 5.65 | 12.7 | 61.9 | 31.48 |
| T2 | 5/10/2019 9:40 | 7/10/2019 13:20 | 25.62 | 29.2 | 26.94 | 52 | 54 | 53 | 6 | 6.65 | 6.27 | 59.6 | 106.92 | 80.9 |
| T3 | 2/10/2019 16:20 | 4/10/2019 14:50 | 25.34 | 27.74 | 26.43 | 48 | 49 | 48 | 6.1 | 6.31 | 6.2 | 73.1 | 96.7 | 83.55 |
| T4 | 4/10/2019 13:40 | 6/10/2019 8:00 | 25.2 | 30.02 | 27.23 | 61 | 62 | 62 | 5.97 | 6.52 | 6.22 | 43.2 | 103.1 | 77.06 |
| T5 | 2/10/2019 12:40 | 4/10/2019 8:40 | 25.39 | 27.71 | 26.25 | 67 | 75 | 68 | 6.37 | 6.9 | 6.56 | 46.1 | 91.2 | 67.91 |
| T6 | 4/10/2019 9:30 | 5/10/2019 13:15 | 25.03 | 29.24 | 27.5 | 52 | 80 | 56 | 6.32 | 8.2 | 6.82 | 75.8 | 120.2 | 99.3 |
| T7 | 6/10/2019 16:30 | 7/10/2019 14:15 | 24.37 | 30.71 | 26.76 | 53 | 55 | 54 | 5.33 | 5.69 | 5.44 | 46.5 | 85.1 | 62.72 |
| T8 | 3/10/2019 11:40 | 5/10/2019 7:45 | 26.35 | 29.24 | 27.69 | 52 | 54 | 53 | 6.33 | 8.2 | 6.9 | 83.2 | 120.2 | 99.69 |
| T9 | 4/10/2019 18:30 | 7/10/2019 11:40 | 26.52 | 28.13 | 27.08 | 57 | 57 | 57 | 6.26 | 6.46 | 6.34 | 75.9 | 92 | 83.12 |
| T10 | 3/10/2019 13:50 | 5/10/2019 8:00 | 24.8 | 29.39 | 26.53 | 56 | 57 | 57 | 6.24 | 6.66 | 6.4 | 40.2 | 97.1 | 65.81 |
| T11 | 3/10/2019 16:50 | 5/10/2019 8:15 | 25.42 | 30.26 | 27.09 | 57 | 59 | 58 | 6.43 | 7.36 | 6.69 | 52.58 | 106.92 | 78.34 |
| T12 | 5/10/2019 13:20 | 7/10/2019 9:30 | 22.95 | 30.14 | 25.91 | 61 | 66 | 64 | 5.9 | 6.25 | 6.02 | 16.5 | 106.4 | 55.76 |
| T1 | 27/06/2020 16:00 | 29/06/2020 16:00 | 20.74 | 25.06 | 21.89 | 49 | 57 | 52 | 5.15 | 5.47 | 5.24 | 11.8 | 71.5 | 29.73 |
| T2 | 23/06/2020 16:30 | 25/06/2020 9:30 | 22.5 | 25.69 | 23.95 | 43 | 46 | 45 | 6.03 | 6.35 | 6.15 | 46 | 88.8 | 67.72 |
| T3 | 24/06/2020 14:45 | 26/06/2020 14:00 | 20.38 | 22.85 | 21.44 | 47 | 48 | 48 | 5.73 | 6.01 | 5.84 | 49.2 | 109.3 | 67.02 |
| T4 | 27/06/2020 16:50 | 29/06/2020 15:10 | 21.3 | 23.19 | 21.93 | 41 | 43 | 42 | 5.77 | 6.07 | 5.88 | 11.6 | 98.2 | 41.5 |
| T5 | 26/06/2020 18:50 | 28/06/2020 9:00 | 21.77 | 24.85 | 22.63 | 65 | 67 | 66 | 5.75 | 5.98 | 5.88 | 55.2 | 84.3 | 67.62 |
| T6 | 23/06/2020 13:30 | 25/06/2020 10:00 | 21.57 | 24.46 | 22.75 | 64 | 79 | 78 | 6.01 | 6.58 | 6.4 | 53.3 | 124.1 | 102.92 |
| T9 | 25/06/2020 14:40 | 27/06/2020 10:15 | 21.74 | 25.54 | 23.02 | 52 | 53 | 52 | 5.92 | 6.42 | 6.06 | 74.4 | 118 | 90.69 |
| T11 | 25/06/2020 13:30 | 27/06/2020 9:50 | 20.6 | 24.31 | 22 | 63 | 65 | 64 | 5.71 | 5.96 | 5.82 | 64 | 99 | 80.52 |

**Note S1**

Evaluating the costs and benefits of agricultural land conversion to wetlands (Interviews)

CONSENT FORM

| Research Team | Jim Smart, Joshua Dyke  Australian Rivers Institute |
| --- | --- |

By signing below, I confirm that I have read and understood the information package and in particular have noted that:

- I understand that my involvement in this research will take the form of a short (approximately 20 minutes) discussion with researchers to help researchers obtain typical costs of constructing and maintaining a wetland, including enablers and barriers to land-wetland conversion in the GBR catchments;
- I have had any questions answered to my satisfaction;
- I understand the risks involved;
- I understand that there will be no direct benefit to me from my participation in this research;
- I understand that my participation in this research is voluntary;
- I understand that if I have any additional questions I can contact the research team;
- I understand that I am free to withdraw at any time, without explanation or penalty;
- I understand that my data will be de-identified and that individual participants will not be identifiable in any reports produced;
- I understand that this research is conducted in accordance with the National Statement on Ethical Conduct in Human Research under Griffith University Research Ethics approval no. GU 2019/941;
- I understand that I can contact the Manager, Research Ethics, at Griffith University Human Research Ethics Committee on 07 3735 4375 (or [[research-ethics@griffith.edu.au](mailto:research-ethics@griffith.edu.au)](mailto:research.ethics@griffith.edu.au)) if I have any concerns about the ethical conduct of the project; and

I agree to participate in the project

| Name |  |
| --- | --- |
| Signature |  |
| Date |  |

## Stakeholder interviews

### Objective

To garner lessons arising from the opinions of stakeholders involved in the Riversdale-Murray Scheme, following more than 15 years’ hindsight.

### Method

Face-to-face semi-structured interviews were conducted with five Riversdale-Murray cane growers who had constructed drainage lagoons on their farm, an agronomist, and an employee of a peak body.

This research was conducted under Griffith University Human Research Ethics approval no. GU 2019/941. Human research ethics approval required data and information obtained to remain anonymous, with reporting not identifiable back to specific data providers or interviewees. The accompanying participant information sheet and consent form are shown in Appendix **Error! Reference source not found.**.

The guiding interview questions were as follows:

- How many years have you been growing sugarcane in in this region?
- Do you also grow other crops as well as sugarcane or carry out other agricultural operations (e.g. cattle fattening) – perhaps at different times of year, or on different areas on your properties?
- What is the typical sugarcane yield from your elevated field(s) in t/ha/yr?
- How much fertiliser do you put on (plant cane and ratoon cane fertiliser rates separately) – and how are fertiliser rates determined?
- From your recollection: what is the average yield; and also highest and lowest yield in tonnes/ha/year on your cane land? What was special about the conditions in those particularly high/low yield years?
- Did you see changes in yield (or usage) on the elevated areas after the wetland was constructed? [What yield did you get from the land before it was elevated? – for how much fertiliser applied?]
  - How consistent is this yield across the years?
  - Are there any weather effects on this difference in yield?
- Does the yield change only affect the elevated area or have other areas of the farm been affected too? e.g. due to improvements from the drainage scheme more generally across the farm
- What about soil properties? Were there any advantages or disadvantages from adding the dredge spoil from the wetland to adjacent fields?
- Are there other area on the farm that yield like the area that was elevated? [y/n] If yes, what yield do you typically get from those areas, for how much fertiliser applied?
- Are there any other benefits to the farm business from constructing the wetland?
  - Trackways are drier, less risk of machinery getting bogged?
  - Benefits for farmer, family and friends from fishing/wildlife watching in the wetland?
- Are any additional costs associated with the wetland?
- What about the on-going maintenance of your farm? Specifically, how much *time* and *money* are required *per year* to maintain your
  - (elevated) sugarcane area to achieve its full productivity
  - Wetland from weed invasion and feral animals
- For your elevated sugarcane growing areas and the creation of a wetland as a result, on your farm
  - What was the nature of the construction/earthworks involved?
  - When was it constructed?
  - How long did it take for the construction work to complete?
  - How many machine hours did it take?
  - Did the construction go according to schedule?
  - How much did the construction work cost in total and what was the breakdown for each major component of costings?
  - Were there any other additional/unexpected construction costs that you had to incur (beyond those initially budgeted e.g. cost over runs)
  - Were maintenance costs for the wetland different to what you expected?
- Looking back, if you had known then what you know now, would you still go ahead and construct the wetland?
- Would you do it again in the future and if so what would be the main drivers of this?
- For the wetland that was created:
  - What is your perception of the effectiveness and benefits of the wetland?
  - What are your views on the barriers and enablers influencing wetland conversion?
- Do you have any other comments you would like to raise?
- Do you know anyone else in the industry who may like to participate in a survey such as this one?


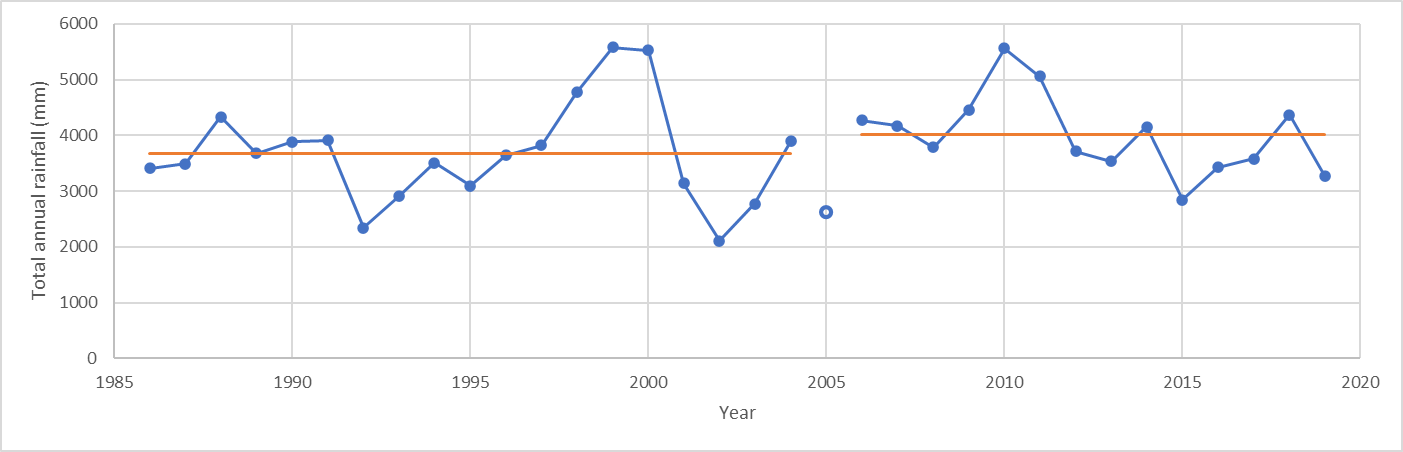
**Figure S1.** Annual rainfall recorded at Tully Sugar Mill (Bureau of Meteorology rainfall recording station number 32042) between 1986 and 2019. Vertical grey bar indicates Scheme construction. Orange lines indicate pre- and post-construction means. Data for 2005 are incomplete, with no rainfall data recorded for December that year.

| **Table S5.** Input parameters for FEAT assessment of a representative farm. Prices and costs are stated in 2019 AUD$, escalated from the default values provided by FEAT in 2015 AUD$ using the Quarterly Consumer Price Index for Brisbane, Queensland (Australian Bureau of Statistics, 2020). | |
| --- | --- |
| **Parameter** | **Value** |
| Area of soil spreading (ha) | 1.25 |
| APSIM soil type | mari |
| APSIM soil permeability | moderate |
| SILO climate zone | 1800_14585 |
| Cane cycle | Plant cane, followed by two or four ratoons and a cow-pea legume fallow. |
| Row spacing | 1.8 m |
| Sugar price | $461/tonne IPS |
| Harvesting cost | $9.11/ tonne |
| Levies | $0.70/tonne |
| Tractor fuel price (after rebate) | $1.07/litre |

| **Table S6.** Estimated wetland cost function based on Equation (1). Dependent variable: *Cost (2003 AUD$)*. | | | |
| --- | --- | --- | --- |
| **Variable** | **Coefficient** | **Robust SE** | **p-value** |
| Constant | 326.628 | 512.844 | 0.528 |
| Volume excavated | 2.501 | 0.206 | <0.001 |
| Volume excavated squared | -0.00004 | 0.000008 | <0.001 |
| Lagoon surface area | -0.708 | 0.212 | 0.002 |
| Number of observations | | 44 | |
| R^2^ | | 0.946 | |
| Adjusted R^2^ | | 0.941 | |
